# Analyzing Peptide Torsional Dynamics: An Angular-Displacement PCA Pipeline for Short-Horizon Prediction from Molecular Dynamics

**DOI:** 10.1021/acs.jpca.5c07760

**Published:** 2026-04-22

**Authors:** Luis Albrizzi, Gabriel Gayoso, José Colbes, Santiago Di Lella, Christian E. Schaerer, Amaury C. Alvarez

**Affiliations:** † Polytechnic School, 187173 National University of Asuncion, Campus de la UNA, Villa Universitaria, Central, P.O.Box: 2111 SL, San Lorenzo 111421, Paraguay; ‡ Instituto de Química Biológica - Ciencias Exactas y Naturales (IQUIBICEN) Conicet - Facultad de Ciencias Exactas y Naturales, 62873 Universidad de Buenos Aires, Ciudad Universitaria, Pab. II, Ciudad de Buenos Aires C1428EHA, Argentina; § Institute of Computing, Federal University of Rio de Janeiro, Av. Athos da Silveira Ramos, 274, Rio de Janeiro 21941-590, Brazil

## Abstract

Analyzing the conformational dynamics of short peptides from molecular dynamics (MD) simulations remains challenging. The high dimensionality of torsional space and the periodic nature of dihedral angles complicate statistical analysis and dimensionality reduction. This work presents an integrated computational workflow that combines all-atom MD simulations with a multistage analytical framework to characterize torsional reorganization patterns. Our approach introduces an angular-displacement representation χ that resolves periodic discontinuities by focusing on frame-to-frame torsional changes rather than absolute configurations. This transformation yields variables suitable for linear analysis and acts as a high-pass filter, emphasizing rapid reorganization events over slow conformational drift. We analyze these transformed coordinates using spatiotemporal principal component analysis (PCA) to identify collective torsional patterns. To evaluate how different coordinate choices preserve dynamical information, we quantitatively compare raw dihedral angles, sine–cosine embedding, and the displacement representation χ using the VAMP score. This comparison reveals their complementary nature: sine–cosine coordinates capture slow conformational variability, while χ highlights rapid torsional reorganizations. Subspace convergence analysis confirms the stability of the reduced PCA representation within the simulation time scale. We apply the methodology to the DENV-2 peptide (CGYGLC) as a representative short system. Our approach identifies hierarchical patterns of torsional flexibility—characterized by a flexible central core and region-specific dynamics—and reconstructs short-term structural evolution with angular errors below 25% and RMSD values of 1.0–2.1 Å. The main contributions are (i) a geometry-aware angular-displacement representation that respects the periodic nature of torsional variables; (ii) a spectral characterization of the displacement transformation; (iii) a quantitative comparison of observable representations using the VAMP score; and (iv) a demonstration of short-horizon structural prediction from reduced dynamical subspaces. The workflow provides a computationally efficient framework for analyzing torsional reorganization dynamics in peptide simulations.

## Graphical abstract

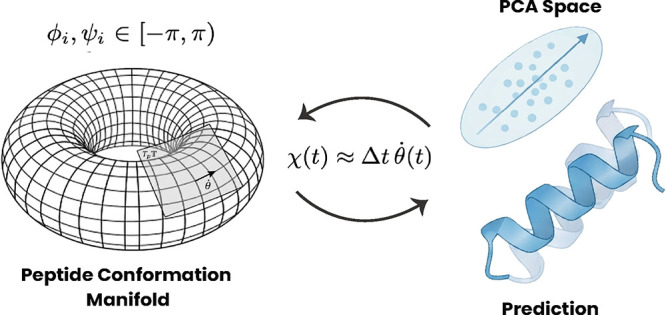

## 1 Introduction

The biological function of proteins and peptides is intrinsically linked to their three-dimensional structure and internal motions. Characterizing how structural variables evolve over time is therefore central to understanding molecular systems from a mechanistic perspective. In short peptides, backbone torsional degrees of freedom provide a compact and information-rich description of conformational variability, making them natural observables for the analysis of molecular dynamics (MD) simulations.

The conformation of a peptide is primarily described by its backbone dihedral angles ϕ and ψ, which determine the accessible regions of the Ramachandran space.^1^ These angular variables provide a compact representation of structural variability, but their periodic nature introduces discontinuities that complicate linear statistical treatments.^2^

Despite advances in structural prediction—notably AlphaFold2 ^3^—the statistical characterization of MD trajectories remains challenging due to high dimensionality, nonlinear torsional geometry, and the coexistence of different dynamical regimes.^4–6^

Here, we characterize torsional reorganizations by analyzing angular displacements between consecutive MD frames, rather than absolute angular configurations.

To this end, we introduce an angular-displacement transform χ,^7^ which resolves periodic discontinuities and represents frame-to-frame changes as geodesic displacements on the torus. In this formulation, χ­(*t*) resides in the tangent space of the angular manifold and describes local reorganizations rather than static conformations.

Fourier analysis of the χ transformation reveals its high-pass filtering characteristics, attenuating slow conformational drift while amplifying rapid torsional events

As an alternative representation, we consider the sine–cosine embedding of dihedral angles, which preserves periodic geometry while retaining information about large-scale angular structure. The ability of each observable set (raw dihedrals, χ, and sine–cosine embedding) to capture slow dynamical modes is quantitatively evaluated using the VAMP score.^8^ This comparison provides an objective criterion for assessing how different coordinate choices preserve slow dynamical structure.

The transformed coordinates are further analyzed through spatiotemporal principal component analysis (PCA) to identify collective torsional reorganization patterns.^2,4^ This reduced description focuses on coordinated angular displacements without imposing discrete state models.

To demonstrate our approach, we applied the workflow to the DENV-2 peptide (CGYGLC) in aqueous solution, using an initial structure from AlphaFold2 ^3^ simulated with AMBER.^9^

To address these challenges, we propose an integrated computational pipeline that combines all-atom molecular dynamics simulations with a multistage analytical framework for characterizing torsional reorganization dynamics. The workflow begins with trajectory generation in AMBER using the ff19SB force field, followed by extraction of backbone dihedral angles ϕ and ψ. A key methodological innovation is the introduction of an angular-displacement representation χ that operates in the tangent space of the torus, resolving periodicity artifacts while preserving the geometric structure of torsional variables. This representation enables linear statistical analyses—including spatiotemporal principal component analysis (PCA) and spectral characterization via Fourier methods—that would otherwise be compromised by angular wrapping. To objectively evaluate the dynamical information captured by different coordinate choices, we employ the VAMP score as a quantitative criterion for comparing raw dihedral angles, sine–cosine embedding, and the displacement representation χ. The reduced dynamical subspace obtained from PCA is then used to explore short-horizon structural prediction through variational dynamical modeling, with prediction quality assessed using angular metrics and RMSD reconstruction. The pipeline is applied to the DENV-2 and TRP-CAGE system, ensuring the robustness of the methodological framework.

The main contributions of this work are (i) a geometry-aware angular-displacement representation (χ) that resolves periodic discontinuities; (ii) a spectral analysis demonstrating the high-pass filtering nature of the χ transformation; (iii) a quantitative comparison of χ, sine–cosine, and raw angle representations using the VAMP score; and (iv) a spatiotemporal PCA that yields a low-dimensional description of collective torsional motions.

The remainder of the article is organized as follows. Section 2 introduces the mathematical representation of the peptide system. Section 3 details the analytical techniques, including angular transformation, spectral analysis, dimensionality reduction, and VAMP-based evaluation. Section 4 presents the characterization of collective torsional modes and representation comparison. Finally, Section 5 discusses methodological implications and limitations.

## 2 Protein Model

We model the conformational dynamics using the dihedral angles θ­(*t*) obtained from molecular dynamics trajectories.^10^ Their evolution is given by

θ(t+τ)=F(θ(t))+η(t) 1

where θ = (ϕ_1_, ···, ϕ_*m*–1_, ψ_1_, ···, ψ_*m*–1_), with ϕ_*i*_, ψ_*i*_ ∈ [−π, π) for all *i* = 1, ···, *m* – 1, and *n* = 2­(*m* – 1), with m∈N denoting the length of the amino acid sequence. The forcing term η­(*t*) accounts for thermal fluctuations, and *F* represents an underlying nonlinear relationship governing conformational changes. To simplify the notation in the article, we write θ_*t*_ = θ­(*t*) and θ_*t*+1_ = θ­(*t* + τ).

In this work, τ = 1 frame = 4 ps. In order to extend the observation horizon, we define the lengths as multiples of τ, i.e., 2τ, 3τ, ···. Furthermore, the indices corresponding to the angles in the images will be enumerated from 1 to 10, so that

$$\theta =(\theta_{1},\cdot \cdot \cdot ,\theta_{10})=(\psi_{1},\phi_{2},\psi_{2},\phi_{3},\psi_{3},\phi_{4},\psi_{4},\phi_{5},\psi_{5},\phi_{6})$$ 2

For a peptide of *m* residues, the angles ϕ_1_ and ψ_*m*_ are not defined due to the lack of adjacent atoms at the N- and C-terminal ends (see Figure 1).

**Figure 1:**
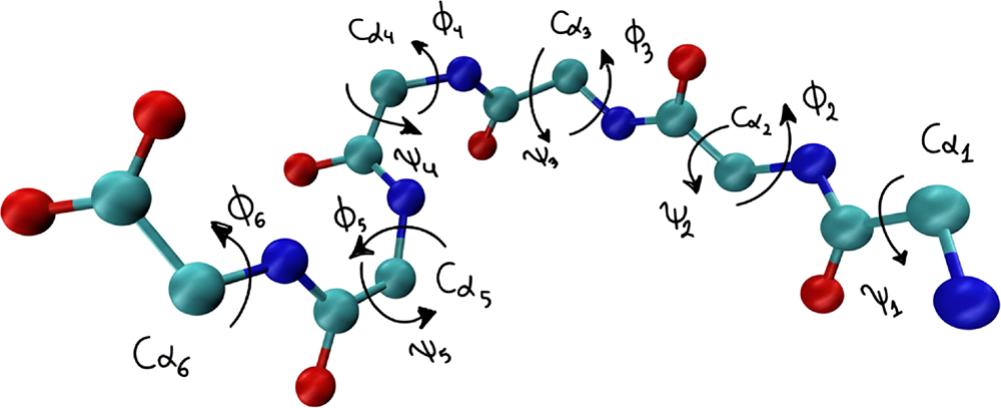
Backbone dihedral angles ϕ_*i*_ and ψ_*i*_ defining the DENV-2 peptide conformation (CGYGLC). The red, sky-blue, and blue spheres represent oxygen, carbon, and nitrogen atoms, respectively. These torsions serve as the fundamental variables for the dynamic analysis. The ten angles analyzed correspond to the following residues: ψ_1_ (Cys1), ϕ_2_, ψ_2_ (Gly2), ϕ_3_, ψ_3_ (Tyr3), ϕ_4_, ψ_4_ (Gly4), ϕ_5_, ψ_5_ (Leu5), and ϕ_6_ (Cys6). As will be shown, the central torsions associated with Gly4 and Leu5 constitute the primary axes of conformational flexibility.

Dihedral angles are computed from the Cartesian coordinates of molecular dynamics simulations.^9^ This representation is particularly useful because torsional angles provide a low-dimensional description of the main conformational variability.^11^ Each dihedral angle is defined by the torsion between four consecutive atomic positions. Specifically, the ϕ angle is defined by atoms C_*i*–1_–N_i_–Cα_*i*_–C_i_, the ψ angle by N_i_–Cα_*i*_–C_i_–N_*i*+1_, and the ω angle by Cα_*i*–1_–C_*i*–1_–N_i_–Cα_*i*_. In this study, we analyze only the ϕ and ψ backbone angles, as they are the primary determinants of secondary structure.

However, because each component of θ_*t*_ is an angular variable, its periodic nature can introduce artificial discontinuities in a linear time-series analysis. For example, a change from 178° to −178° represents a true variation of only 4° but appears as a large jump of 356°.^2^

A common strategy to overcome this issue is to embed each dihedral angle into a two-dimensional representation using sine and cosine transformations, replacing θ by the pair (sin θ, cos θ). This embedding removes the discontinuities associated with periodic boundaries but increases the dimensionality of the representation from *d* torsional variables to 2*d* features.^12^ The sine–cosine representation has therefore been widely adopted in dimensionality reduction and conformational analyses of peptides and nucleic acids.^2,12^

However, this mapping places the data on a curved manifold (the torus), which can complicate linear statistical treatments and obscure the interpretation of collective motions in terms of the original torsional degrees of freedom. Here, we explore an alternative representation based on angular displacements χ, which operates directly in the tangent space of the torus. As we will show, this formulation highlights distinct dynamical regimes—particularly rapid torsional reorganizations—and provides a complementary perspective to the sine–cosine embedding, enabling the identification of coordinated dynamical events that are less accessible through conventional angular encodings.

To address discontinuities in angular time series, we apply a standard unwrapping procedure that converts each dihedral angle θ_*t*_^(*j*)^ ∈ [−π, π) into a continuous trajectory $\gamma_{t}^{\left(j\right)}\in R$ by adding multiples of 2π whenever a jump larger than π is detected.^7^ This transformation, denoted *U*, is reversible: applying the modular projection *M*(γ) = (γ + π) mod 2π – π recovers the original angles. A formal proof of reversibility is given in the Supporting Information (Section S1).

Once the unwrapped trajectory **γ**_*t*_ is obtained, we define the instantaneous angular displacement

$$\chi_{t}=\gamma_{t}-\gamma_{t-1}=M(\theta_{t}-\theta_{t-1})$$

which captures the true physical change between consecutive frames without the artifacts of angular periodicity. This quantity, denoted χ, serves as the primary input for subsequent linear analyses.

The relationship between the wrapped angles θ_*t*_, the unwrapped coordinates γ_*t*_, and the displacements χ_*t*_ is schematized in Figure 1-S1 of the Supporting Information. In essence, the unwrapping operation removes 2π discontinuities, yielding a continuous path γ_*t*_ in $R^{n}$ ; the displacement χ_*t*_ is then the difference between consecutive frames. The inverse mapping χ^–1^ reconstructs γ_*t*_ from the displacements, and the modular projection *M* returns to the original torus. This reversible pipeline ensures that any analysis performed on the linearized variables can be translated back to the physically meaningful dihedral angles.

## 3 Model Dynamics

This section describes the mathematical techniques used to analyze the DENV-2 dynamics. We use principal component analysis (PCA) to analyze which angles are more important in accordance with their contribution to the motion of the protein, and the regions of larger spatiotemporal variability^13^ in the peptide in terms of the angles χ. This helps to identify the angles with the largest variability and perform a model reduction (see Section 3.2). The protein’s dynamics using the PCA reduced model is also discussed (Section 3.5).

### 3.1 Observable Selection for Koopman Approximation

A key challenge in Koopman-based analysis is selecting observables that yield accurate linear representations of the nonlinear dynamics. In this work, we systematically evaluate three distinct observable representations:^8,14,15^

1. **Raw dihedral angles** (θ): The native periodic coordinates that directly encode backbone conformation but suffer from wrapping discontinuities.
2. **Angular displacement** (χ): The unwrapped differential representation that provides continuous trajectories while preserving physical interpretability.
3. **PCA projections** (*y*): The reduced-dimensional coordinates that capture maximal variance and collective motions.

Each representation offers different trade-offs between physical interpretability, mathematical tractability, and predictive performance. The χ representation is particularly advantageous as it resolves periodicity issues while maintaining a direct connection to conformational changes. The PCA representation *y* provides optimal dimensionality reduction but may obscure residue-specific interpretations.

We employ the Variational Approach for Markov Processes (VAMP) as a principled framework for comparing these observable sets. The VAMP-2 score quantifies how well each representation captures the slow dynamical modes, while prediction errors and angular metrics assess their practical utility for forecasting. This systematic comparison allows us to identify the most robust observable set for capturing DENV-2’s conformational dynamics while providing general insights into observable selection for peptide systems.

### 3.2 Dimension Reduction

We analyze the angle dynamics of the DENV-2 peptide using principal component analysis (PCA) to identify the directions of greatest variability in both time and space. We collect all displacement vectors χ_*t*_ (*t* = 1, ···, *T*) into a data matrix $X\in R^{T\times n}$ (with *n* = 10 dihedral angles). Principal component analysis (PCA) is performed via singular value decomposition:

$$X=U\Sigma V^{T}$$

where the columns of *V* contain the spatial principal components (PCs) (see Section S2), and the columns of *U*Σ (denoted *y*_*k*_) are the corresponding temporal components. The eigenvalues λ_*j*_ = Σ_*jj*_^2^ indicate the variance captured by each mode. We retained the number of principal components explaining at least 80% of the total variance. This reduction allows us to focus on the dominant collective motions of the peptide.

### 3.3 Determination of the Analysis Frequencies of the Filtering

Projecting *X* onto its principal components yields the temporal scores *Y* = *XV* = *U*Σ, whose columns *y*_*k*_ represent the time evolution of each mode.

Whenever there is a significant change in the angles, a peak appears in the temporal PCA components *y*_*k*_. These peaks indicate moments of strong collective torsional reorganization in the system. To isolate the most relevant dynamical events and suppress small fluctuations associated with noise, we apply a thresholding procedure to the temporal series *y*_*k*_.

This restriction is determined using a specific threshold ϵ. If |*y*_*k*_| > ϵ, the value is preserved as *y̅* ^*k*^ = *y*_*k*_; otherwise it is set to zero, i.e.,

$${\mathrm{y̅}}_{k}^{j}:=\{\begin{array}{ll} y_{k}^{j}, & \mathrm{if}\left|y_{k}^{j}\right|>\epsilon \\ 0, & \mathrm{if}\left|y_{k}^{j}\right|<\epsilon . \end{array}$$ 3

The threshold ϵ is chosen so that a fixed percentage λ_*k*_ *%* of the values *y*_*k*_^*j*^ lie outside the interval [−ϵ, ϵ]. More specifically, the absolute values |*y*_*k*_^*j*^| are sorted, and ϵ is set as the value at the (100 – λ_*k*_)-th percentile. This guarantees that the filtered signal *y̅*_*k*_ captures the most significant fluctuations in the temporal mode *y*_*k*_, highlighting the dominant collective reorganizations while removing small-amplitude variations. The threshold ϵ is chosen to preserve the most significant fluctuations while balancing model sensitivity and robustness against noise.

To characterize patterns of collective torsional reorganizations, we discretize the temporal activity of the PCA modes. At each time step, we examine which of the first *r* temporal components *y*_*k*_ exceed a threshold ϵ (chosen to retain only significant fluctuations). A label $l_{t}$ is assigned according to the activity pattern of the components:

-
$l_{t}=0$ if no component is active;
-
$l_{t}=k$ if exactly one component *k* is active;
-
$l_{t}=r+1$ if two or more components are simultaneously active.

This procedure produces a finite set of activity patterns *S* = {0, 1, ···, *r*, *r* + 1}, each representing a distinct configuration of collective mode activation.

To analyze how these activity patterns evolve over time, we count the observed transitions between labels throughout the trajectory. For each pair (*i*, *j*), we record how often the system moves from pattern *i* to pattern *j* and normalize these counts to obtain a transition frequency matrix $P\in R^{\left|S|\times |S\right|}$ .

The element *P*_*ij*_ therefore represents the relative frequency with which the activation pattern *i* is followed by pattern *j* in the trajectory. This representation provides a coarse statistical description of how collective torsional reorganizations propagate in time.

### 3.4 Conformational States Identification

To identify possible conformational states, we model the trajectory as a Markov process {ζ_*t*_}_*t* = 0_^*T*^ with a fixed lag time τ. The evolution of the system is characterized by a transition density ρ­(ζ_*t*_, ζ_*t*+1_), which defines the conditional probability density of transitioning from state ζ_*t*_ to state ζ_*t*+1_ after a lag τ.

The observable, denoted ζ, can represent several forms of input data, including the raw dihedral angles (θ), the function (χ), or the projections onto principal components (*y*). Throughout this section, we use the general notation ζ_*t*_ to denote the time series of these observables, specifying their origin when necessary.

Although the dynamics are inherently nonlinear, we seek a linear approximation in the feature space via the Koopman operator theory. That is, we aim to identify a linear operator **K** such that the evolution of observables is approximately linear in expectation:

$$E[\zeta_{t+1}(x)]\approx K^{T}E[\zeta_{t}(x)]$$ 4

where **x** denotes the state variables, and **K** is the Koopman matrix approximating the time evolution in the observable space.

To compute **K**, we first define the following time-lagged covariance matrices:

$$C_{00}=E_{t}[\zeta_{t}(x)\zeta_{t}(x{)}^{T}]$$ 5

$$C_{01}=E_{t}[\zeta_{t}(x)\zeta_{t+1}(x{)}^{T}]$$ 6

$$C_{11}=E_{t+1}[\zeta_{t+1}(x)\zeta_{t+1}(x{)}^{T}]$$ 7

where $E_{t}$ and $E_{t+1}$ represent expectations over time and lagged time steps, respectively.

The Koopman matrix **K** is obtained as the solution to the following least-squares minimization problem:

$$\min_{K}\;E[∥\zeta_{t+1}(x)-K^{T}\zeta_{t}(x){∥}^{2}]$$ 8

whose analytical solution is

$$K=C_{00}^{-1}C_{01}$$ 9

With respect to the state variable **x** used in the Koopman operator above, it can take two forms. When ζ = θ, state variables correspond to the Cartesian coordinates of each atom, denoted by **r**; and when ζ = χ, the state variables correspond to the dihedral angles of the macromolecule, denoted by θ. Therefore, **x** = **r** or **x** = θ, depending on the case.

We can approximate the future state at time *t* + τ using the formulation proposed in the Deeptime article.^15^ This approximation is given by the following equation:

$${\hat{\zeta }}_{t+\tau }=(\Phi^{T}{)}^{-1}\Lambda \Psi^{T}(\zeta_{t}-\mu_{0})+\mu_{t}$$ 10

where Φ, Λ, Ψ correspond to the right-singular vectors, singular values, and left-singular vectors, respectively, obtained from the singular value decomposition of the transition operator approximating the evolution from ζ_*t*_ to ζ_*t*+τ_.

In some cases, a scaling factor is applied to the VAMP predictions so that their overall magnitude matches that of the true signal at time *t* + τ. The optimal scaling factor α is determined by minimizing the difference between the Frobenius norms of the scaled prediction and the true data:

$$\min_{\alpha }|∥\alpha {\hat{\zeta }}_{t+\tau }{∥}_{F}-∥\zeta_{t+\tau }{∥}_{F}|$$ 11

This condition is satisfied when both norms are equal, leading to the following expression for the scaling factor:

α=∥ζt+τ∥F∥ζ^t+τ∥F 12

This simple rescaling adjusts the magnitude of the prediction without introducing additional fitting parameters.

### 3.5 PCA Dynamics

The projection of the variables $\chi_{t}\in R^{n}$ onto the PCA space is given by

$$y_{t}=\chi_{t}V$$ 13

where $V\in R^{n\times r}$ contains the first *r* principal components, assumed orthonormal (i.e., *V* ^*T*^ *V* = *I*), and χ_*t*_ are the original observables. Consequently, the time derivative of *y*_*t*_ is

$${\dot{y}}_{t}={\dot{\chi }}_{t}V$$ 14

Consequently, a linear model in the χ space (e.g., from VAMP) induces a consistent linear model in the reduced PCA subspace: $E[y_{t+1}]\approx E[y_{t}]K_{y}$ , where *K*_*y*_ = *V* ^*T*^ *KV* (see Supporting Information, Section S2). This relation shows that any linear model in the original observable space induces a consistent linear model in the PCA subspace, allowing efficient reduced-order prediction.

### 3.6 Angular Metric

The VAMP method, applied to various forms of the variable ζ (such as the angles θ and χ, and the coordinate *y*), enables single-step (unistep) predictions across multiple lag times τ. This approach exhibits relatively low error under specific structural conditions of the macromolecule being studied. Furthermore, we observe a meaningful correspondence between the leading VAMP components and the system’s underlying dynamical modes, enabling the reconstruction of key dynamical features.

The procedure for comparing predictions made in different representations is illustrated in Figures 2 and 3 of the Supporting Information. In summary, for each representation (θ, χ, or *y*), the corresponding VAMP predictor produces a one-step forecast. Predictions originating in χ or *y* are transformed back to angular space via the inverse mappings χ^–1^ and *M* (or first $V^{T}$ then χ^–1^ and *M*). The reconstructed angles *θ̂* are then compared to the true reference angles θ using the angular distance defined below. This ensures that all models are evaluated on the same physical observable.

To quantify the similarity between the real and predicted angular trajectories, we used the Euclidean angular metric on the torus $T^{n}$ , which is appropriate for variables defined modulo 2π, such as dihedral angles. For two angle vectors $\theta ,\hat{\theta }\in T^{n}$ , the per-component shortest angular difference is

$$d(\theta_{j},{\hat{\theta }}_{j})=\min(|\theta_{j}-{\hat{\theta }}_{j}|,2\pi -|\theta_{j}-{\hat{\theta }}_{j}|)$$

The overall distance for a given frame is the Euclidean norm of these component-wise differences, normalized by the maximum possible distance $d_{\max}=180\sqrt{n}$ (degrees). Thus, the relative error per frame is

$$e=\frac{1}{d_{\max}}\sqrt{\sum_{j=1}^{n}d(\theta_{j},{\hat{\theta }}_{j}{)}^{2}}\in [0,1]$$

Although VAMP can be applied iteratively for multistep forecasting, we find that recursive usage often leads to a rapid accumulation of prediction error. Therefore, we adopt a single-step strategy in which the lag time τ is progressively increased until the resulting error remains within a predefined tolerance. This tolerance depends on both the structural complexity of the macromolecule and the desired level of predictive accuracy. The accumulation error at the lag time τ is given by

$$\langle e(\tau )\rangle =\frac{1}{N}\sum_{k=1}^{N}e_{k}$$

where *N* is the number of validation samples.

When the PCA representation is used, we denote by y* the angular trajectories reconstructed from the PCA coordinates *y* through the inverse mappings $V^{T}$ , χ^–1^, and *M*, as illustrated in Figure 3 of the Supporting Information. In this case, predictions are compared against these reconstructed angular trajectories rather than against the original θ trajectory. This procedure isolates the reconstruction error introduced by the dimensionality reduction and allows the predictive performance in the reduced PCA subspace to be evaluated independently of the projection step.

### 3.7 Methodological Pipeline

The methodology is summarized in Algorithm 1, which includes all the steps defined throughout the paper.
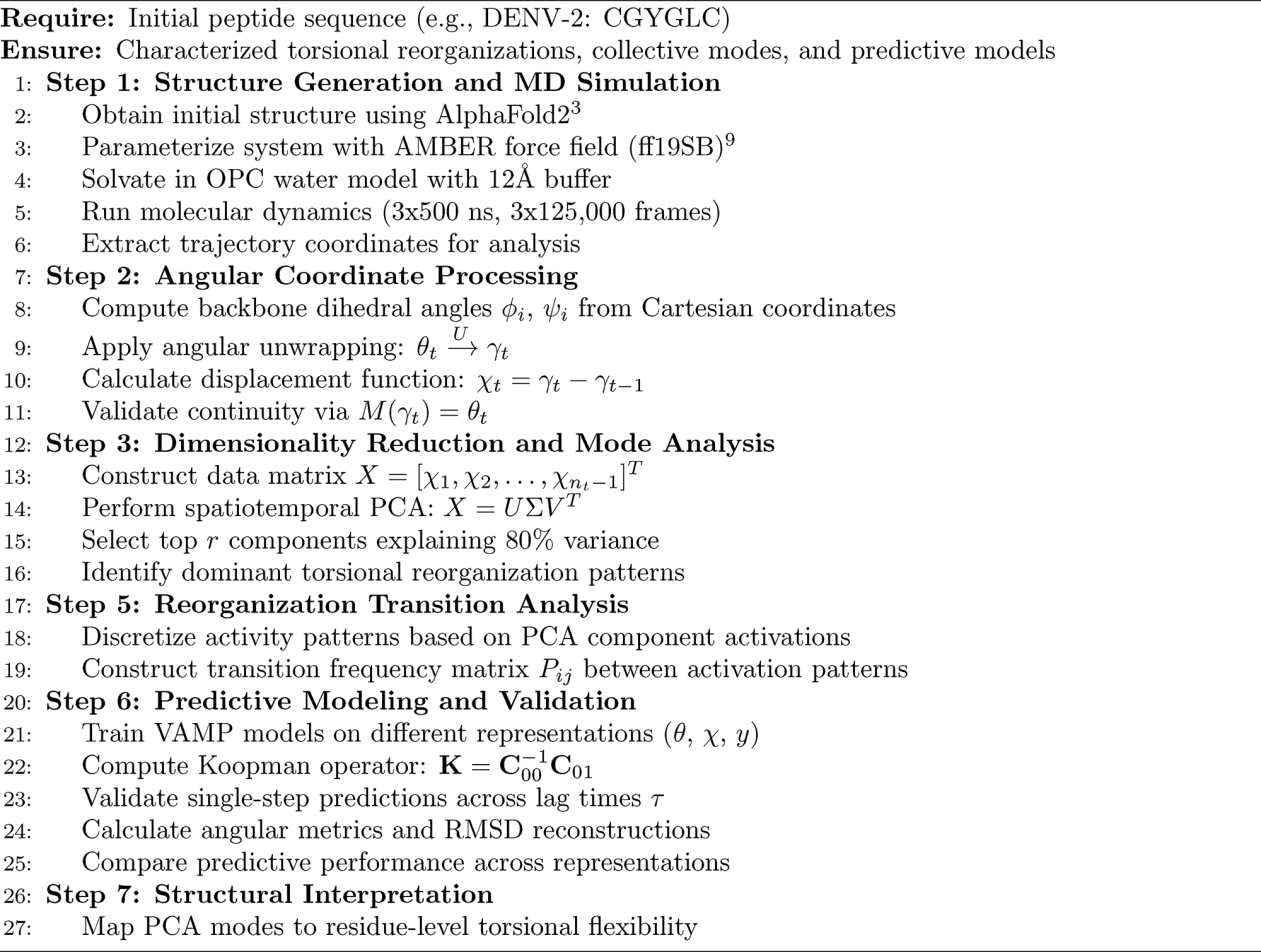


## 4 Experimental Results

In this section, we present the results obtained by applying the proposed methodology to the DENV-2 peptide. First, we evaluate the dynamical representations in terms of stability, spectral content, and their ability to preserve relevant dynamical information. Next, we analyze the dominant spatial and temporal patterns identified by the PCA decomposition, together with the transition structure between activation patterns. Finally, we assess the predictive performance of the VAMP-based models using angular error metrics and RMSD reconstruction.

### 4.1 Observable Selection for Koopman Analysis

Our systematic comparison of observable representations (θ, χ, *y*) reveals important considerations for Koopman-based modeling of biomolecular systems. While the raw dihedral representation θ achieved the lowest prediction errors in angular space, it requires careful handling of periodicity and may not be optimal for all applications. The displacement function χ provides an excellent balance, resolving mathematical discontinuities while maintaining physical interpretability. The PCA representation *y*, though losing some residue-specific information, offers the most compact representation and best captures collective motions.

Our results show that the optimal observable depends on the analysis goal. The χ representation is most suitable for residue-level interpretation, the PCA representation *y* provides the most compact description of collective motions, and the raw angles θ yield the highest short-term predictive accuracy in angular space. The VAMP framework proved invaluable for this comparison, providing a principled metric for evaluating how well each representation captures the essential dynamics.

### 4.2 Results of the Application of the Method to DENV-2

#### 4.2.1 Validation of the Dynamical Representation

To evaluate the stability of the reduced torsional representation, we analyze how the PCA subspace evolves during the first 100 ns of simulation. Specifically, we compare the principal components obtained from partial trajectories with those computed from the full 500 ns trajectory.

To quantify this similarity, we compute the normalized subspace overlap

$$S(t)=\frac{1}{r}∥V(t{)}^{T}V(T){∥}_{F}^{2}$$ 15

where *V* (*T*) contains the first *r* principal components obtained from the full trajectory and *V* (*t*) the components computed using the trajectory truncated at time *t*.

The quantity *S*(*t*) measures the alignment between both PCA subspaces and takes values between 0 and 1. Values close to 1 indicate that the reduced dynamical subspace obtained at time *t* is essentially identical to that obtained from the complete simulation.

Figure 2 shows the evolution of *S*(*t*) for the first five principal components across three independent replicas. In all cases, the overlap increases rapidly during the first tens of nanoseconds and approaches values very close to unity (*S*(*t*) > 0.995) after approximately 30–40 ns. This behavior indicates that the dominant torsional reorganization subspace stabilizes early in the trajectory and remains consistent throughout the simulation. The close agreement between the three replicas further demonstrates that the reduced PCA representation is robust with respect to sampling variability. Consequently, the dominant collective torsional modes identified by PCA reflect intrinsic dynamical patterns of the peptide rather than trajectory-specific fluctuations.

**Figure 2:**
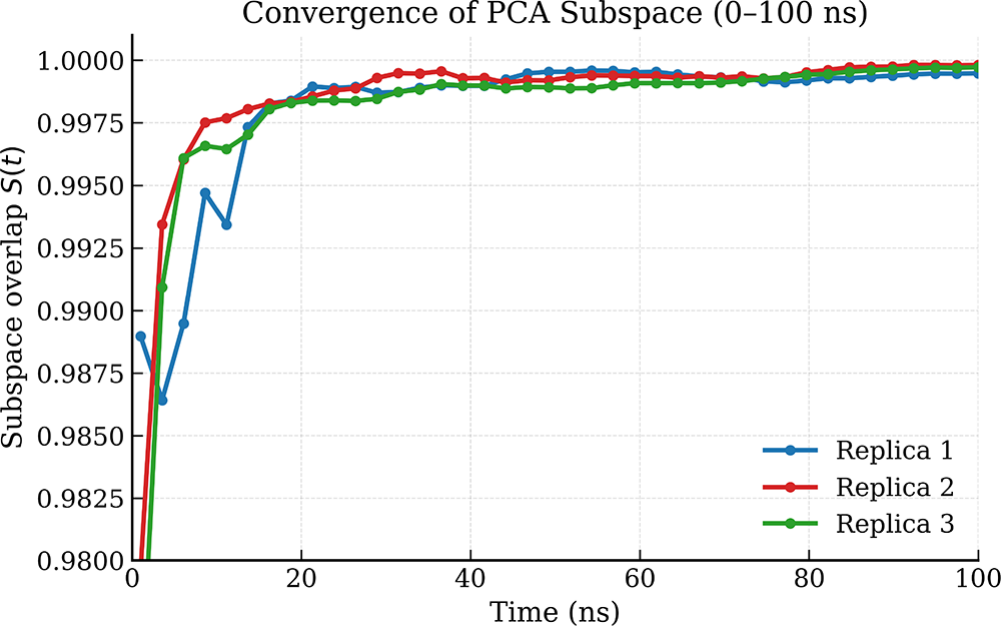
Time evolution of the normalized PCA subspace overlap $S(t)=\frac{1}{r}∥V(t{)}^{T}V(T){∥}_{F}^{2}$ for the first *r* = 5 principal components. The overlap quantifies the alignment between the reduced subspace computed from partial trajectories and that obtained from the full 500 ns data set. The rapid convergence of *S*(*t*) toward unity suggests early stabilization of the dominant torsional reorganization subspace and is consistent across independent replicas.

To compare the dynamical content preserved by different observable representations, we compute the VAMP-2 score as a function of lag time τ. The score is evaluated for three representations: (i) raw dihedral angles θ, (ii) sine–cosine embedding, and (iii) angular displacement χ.

Figure 3 shows the evolution of the VAMP-2 score for increasing lag times. As shown in the figure, the sine–cosine embedding achieves the highest VAMP-2 scores across all lag times, indicating that this representation preserves the largest amount of slow dynamical variance. In contrast, the raw angular representation (θ) yields intermediate scores, while the displacement representation (χ) exhibits substantially lower values.

**Figure 3:**
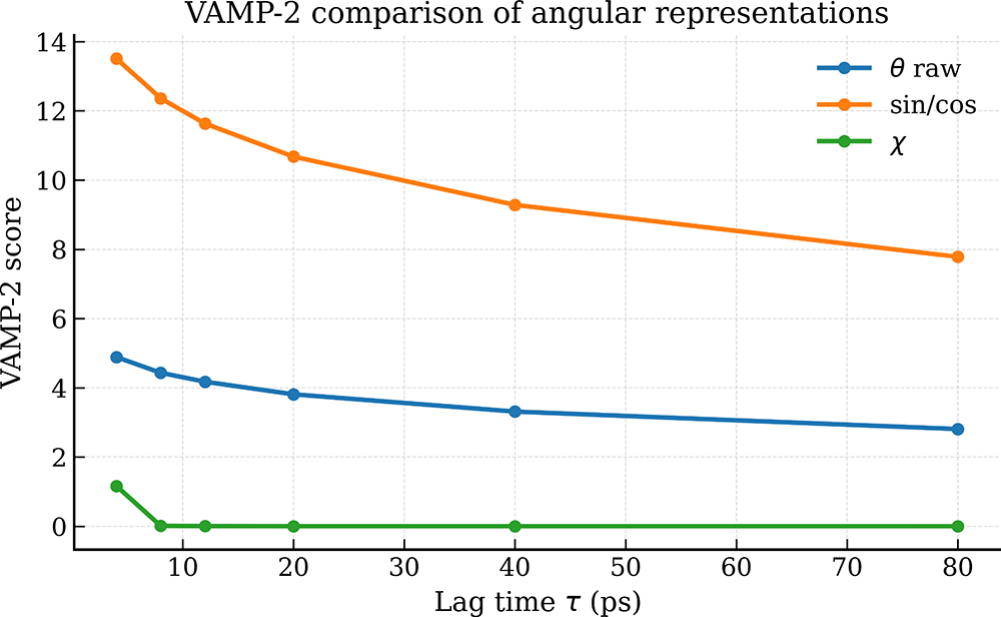
VAMP-2 score as a function of lag time τ for raw dihedral angles (θ), sine–cosine embedding, and angular displacement representation (χ). The score quantifies the dynamical variance retained at each lag time for the corresponding representation.

This behavior aligns with the interpretation of χ as a measure of frame-to-frame variation. By capturing rapid torsional reorganizations, it does not maximize the VAMP score, which is designed to identify slow dynamical processes.

Taken together, these results indicate that the three representations emphasize complementary dynamical regimes: sine–cosine coordinates capture slow conformational variability, raw angles preserve overall structural information, and the displacement representation highlights rapid torsional reorganization events.

##### Spectral Characterization

To examine the frequency content of the angular representations, we compute the power spectral density (PSD) using Welch’s method^16^ for both the raw dihedral signal and the angular displacement representation.

Figure 4 reveals a clear redistribution of spectral energy between the two representations. The raw angular signal (θ) concentrates most of its spectral power at low frequencies, reflecting slow conformational fluctuations and long-time scale structural drift.

**Figure 4:**
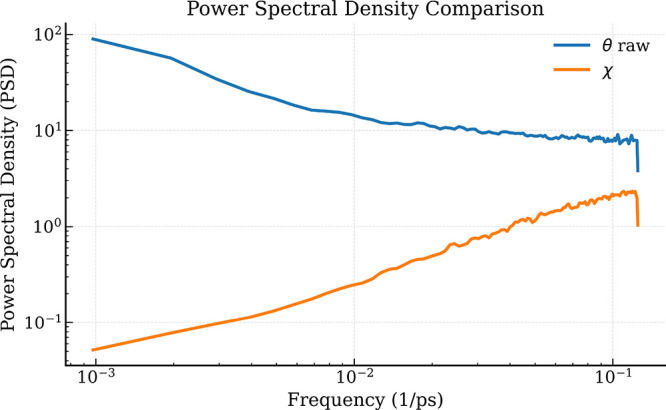
Power spectral density (PSD) of a representative angular coordinate for the raw dihedral signal (θ) and the displacement representation (χ), computed using Welch’s method. The spectra are shown on a log–log scale to highlight differences across frequency ranges.

In contrast, the displacement representation (χ) shifts spectral energy toward higher frequencies. This behavior arises because χ corresponds to the discrete difference of the unwrapped angular trajectory, which acts as a high-pass transformation that attenuates slow variations while amplifying rapid changes.

As a result, the χ representation emphasizes fast torsional reorganizations and transient dynamical events, whereas the raw angles capture the slower conformational evolution of the peptide. This spectral separation provides a mechanistic explanation for the differences observed in the VAMP analysis.

##### High-Frequency Energy Fraction

To quantify the redistribution of spectral energy across frequency ranges, we compute the fraction of total spectral energy contained above a cutoff frequency *f*_c_.

For each representation, this quantity is defined as

$$R(f_{c})=\frac{\int_{f>f_{c}}\mathrm{PSD}(f)df}{\int \mathrm{PSD}(f)df}$$ 16

which measures the proportion of total spectral energy located in the high-frequency regime.

Figure 5 shows *R*(*f*_c_) as a function of the cutoff frequency for both the raw angular signal and the displacement representation. The vertical dashed line marks the reference cutoff *f*_c_ = 0.02 (1/ps).

**Figure 5:**
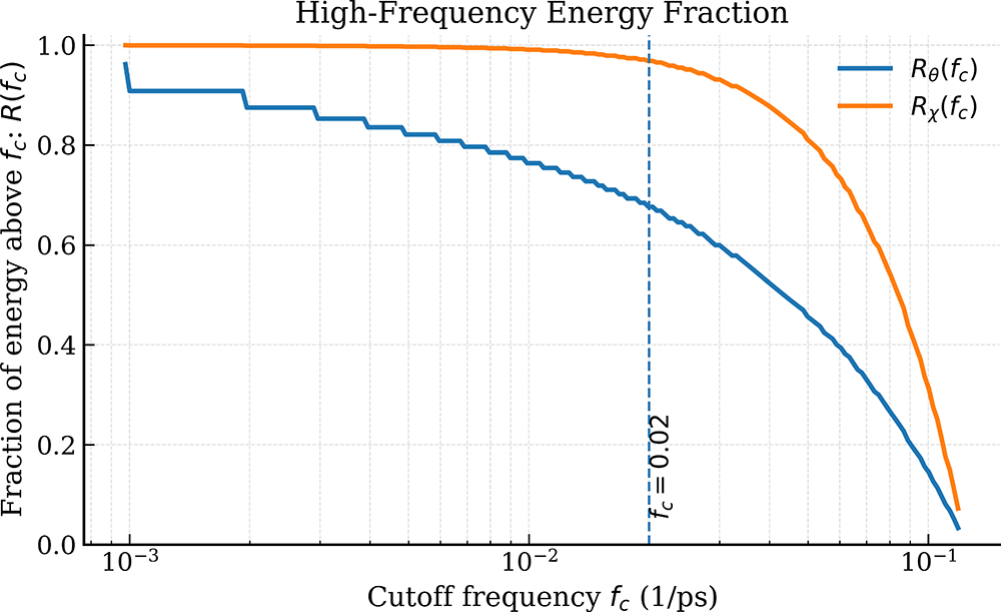
Fraction of spectral energy contained above the cutoff frequency *f*_c_ for the raw angular signal (*R*_θ_) and the displacement representation (*R*_χ_). The curves are obtained by integrating the power spectral density above *f*_c_ and normalizing by the total spectral energy. The dashed line indicates the reference cutoff *f*_c_ = 0.02 (1/ps).

Figure 5 quantifies the redistribution of spectral energy across frequency ranges. For all cutoff frequencies, the displacement representation (*R*_χ_) retains a substantially larger fraction of high-frequency energy than the raw angular signal (*R*_θ_), that is,

$$R_{\chi }(f_{c})\gg R_{\theta }(f_{c})$$ 17

At the reference cutoff *f*_c_ = 0.02 (1/ps), the fraction of energy above the cutoff remains close to unity for the displacement representation, while the raw angular signal retains a significantly smaller fraction. This confirms that the χ transformation strongly amplifies fast dynamical components relative to the original angular coordinates (see Figure 5).

These results support the interpretation that the displacement representation acts as a dynamical filter that highlights rapid torsional reorganizations, complementing the slower conformational information captured by the raw angular representation.

#### 4.2.2 Spacial Components

We note that the native DENV-2 peptide is cyclized via a disulfide bridge between its two cysteine residues. In this study, we deliberately simulated the linear form of the peptide to evaluate the performance of our workflow on a highly flexible, unconstrained system. This choice allows us to demonstrate that the methodology is applicable to peptides regardless of their cyclization state, and that it can capture the full range of torsional reorganizations in the absence of covalent constraints. The linear form therefore serves as a stringent test case for the angular-displacement representation and the subsequent dynamical analysis.

The spatial PCA analysis of the DENV-2 peptide reveals that the first five principal components (PCs) account for approximately 82.1% (see Figure 6) of the total structural variability, confirming that a reduced subset of angular modes captures most of the system’s conformational motion.^4^ This variance concentration in the first few modes suggests that the peptide’s dynamics are highly correlated, and that a reduced set of coordinates can describe the dominant conformational changes.

**Figure 6:**
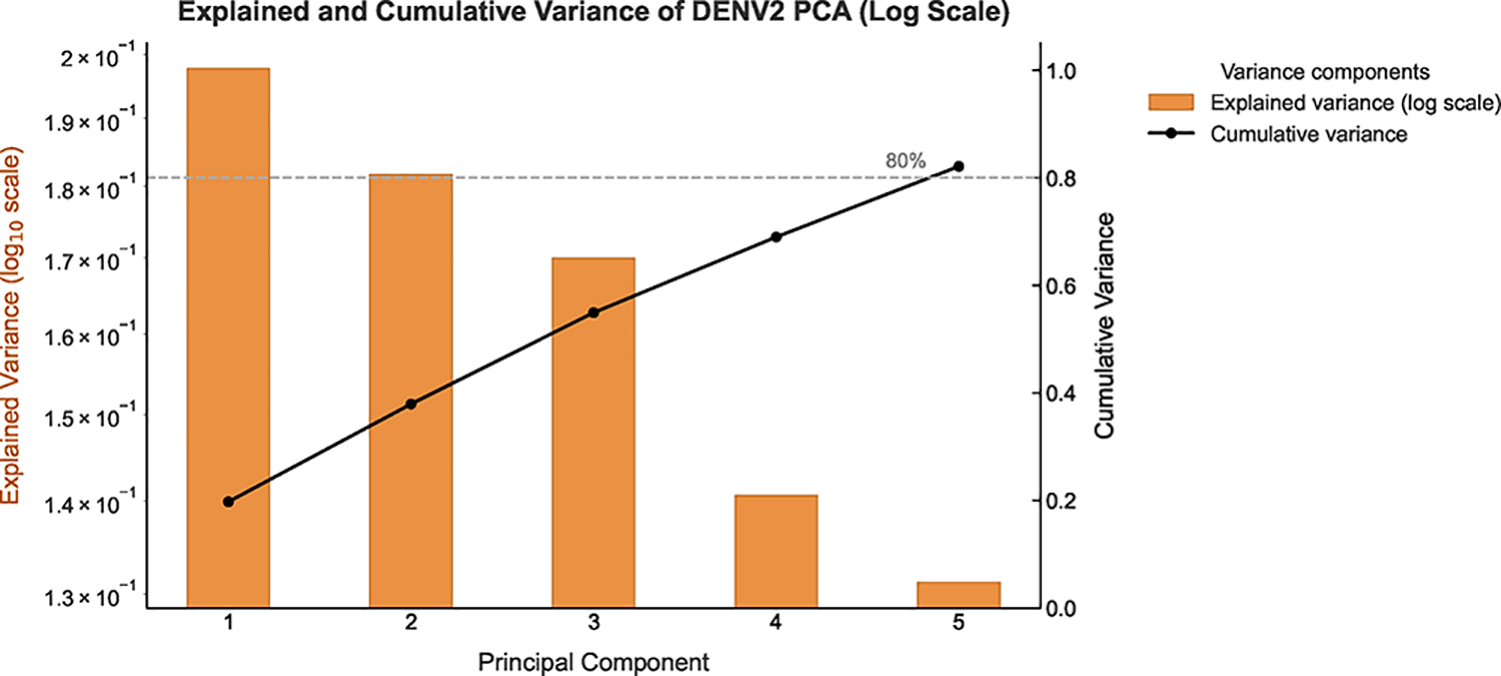
Explained (log scale) and cumulative variance of the principal components for the DENV-2 system. The first five PCs account for more than 80% of the total variance, demonstrating the effectiveness of dimensionality reduction.

Figure 7 reveals the most significant contributions of the dihedral angles to the first five principal components of the system. In this interpretation, only the most intense contributions—represented by the reddest (positive) and bluest (negative) colors—are considered, in order to identify the angles responsible for the dominant collective oscillations and their structural involvement.

**Figure 7:**
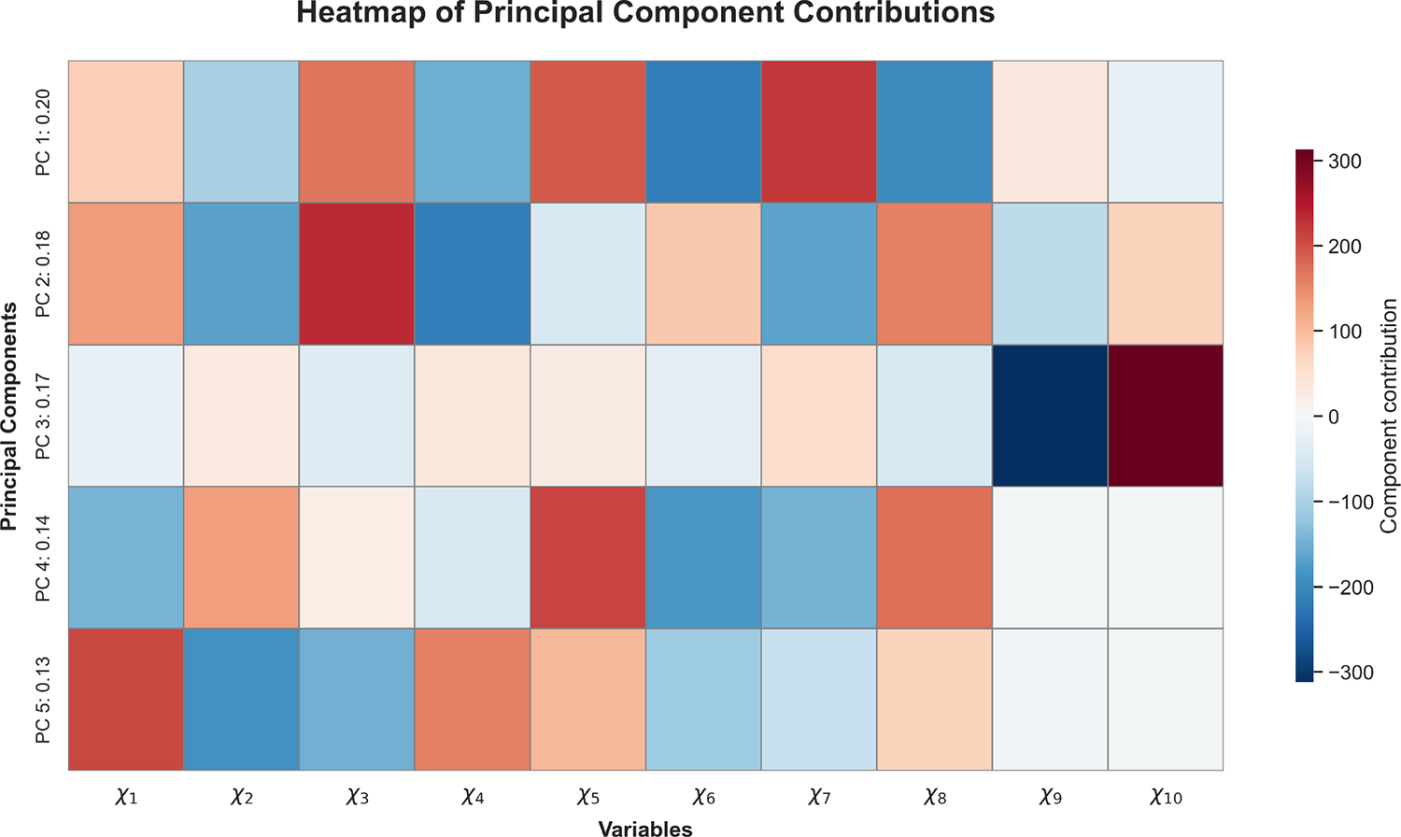
Heatmap of the five dominant spatial principal components (PCs) for the DENV-2 peptide (CGYGLC). Red and blue indicate positive and negative torsional contributions, respectively. The analysis reveals a highly flexible core localized to the central Tyr–Gly–Leu (YGL) segment, driven primarily by the high-amplitude motions of torsions ϕ_4_ (χ_6_), ψ_4_ (χ_7_), and ϕ_5_ (χ_8_) across multiple PCs. In contrast, the terminal Cys residues exhibit more localized and selective movements, such as the scissor-like motion at the C-terminus captured by PC3. This pattern indicates a structured distribution of torsional flexibility, with a dynamically active central region flanked by comparatively constrained termini.

The spatial PCA decomposition reveals structured patterns of angular contributions that differentiate between central and terminal regions of the DENV-2 peptide.

Notably, angles ϕ_4_, ψ_4_, and ϕ_5_ (angles 5–7), located in the central region, exhibit consistent and high-amplitude activations across multiple principal components (PCs), particularly in modes 1, 2, 4, and 5. This suggests that these central torsions constitute a core of conformational flexibility, acting as primary axes of collective motion.

In contrast, the terminal angles display more localized and mode-specific behaviors. The C-terminal angles ψ_5_ and ϕ_6_ (angles 9 and 10) are selectively activated in mode 3 with opposing signs, indicating a scissor-like motion restricted to the terminal region. The N-terminal angles (ψ_1_, ϕ_2_) participate predominantly in modes 2, 4, and 5, where alternating signs reflect phase-inverted oscillations.

From the row-wise interpretation, each mode captures distinct patterns of collective oscillations. Mode 1 exhibits anticorrelated dynamics in the central segment (angles 2 to 8), indicating a compensatory mechanism spanning consecutive torsions. Mode 2 combines terminal and central activations with opposing signs, suggesting long-range compensatory effects. Mode 3 isolates C-terminal movement, while mode 4 represents coherent motion in the central region without sign inversion. Mode 5 reflects synchronized oscillations across adjacent angles, pointing to a cooperative structural wave.

Overall, the combined row- and column-wise analysis of Figure 7 reveals that the peptide’s angular dynamics are organized around a highly flexible central core and dynamically decoupled terminal regions. The principal components isolate distinct structural mechanisms: phase-inverted torsional modes (e.g., modes 1 and 3), compensatory alternation (mode 2), and coherent oscillatory coordination (mode 5). These results support the hypothesis that conformational flexibility is not homogeneously distributed, but rather structured into robust collective patterns likely relevant to the peptide’s antiviral function.

#### 4.2.3 Temporal Components

To characterize the temporal evolution of torsional reorganizations, we considered transitions between discrete activation patterns defined in the PCA temporal space. Specifically, the dynamics were partitioned into 7 patterns according to the activation of the temporal principal components: one baseline pattern with no significant activation (Pattern 0), five single-component activation patterns (Patterns 1–5), and one multicomponent coactivation pattern. This discrete 7-pattern representation provides the basis for the subsequent transition analysis.

Figure 8 shows the transition structure obtained from the activation patterns of the principal components. The transition matrix and its graphical representation reveal that the most persistent patterns correspond to the baseline configuration (no dominant activation) and to the simultaneous coactivation of multiple components. In contrast, patterns associated with isolated component activations are typically short-lived and appear as intermediate dynamical events.

**Figure 8:**
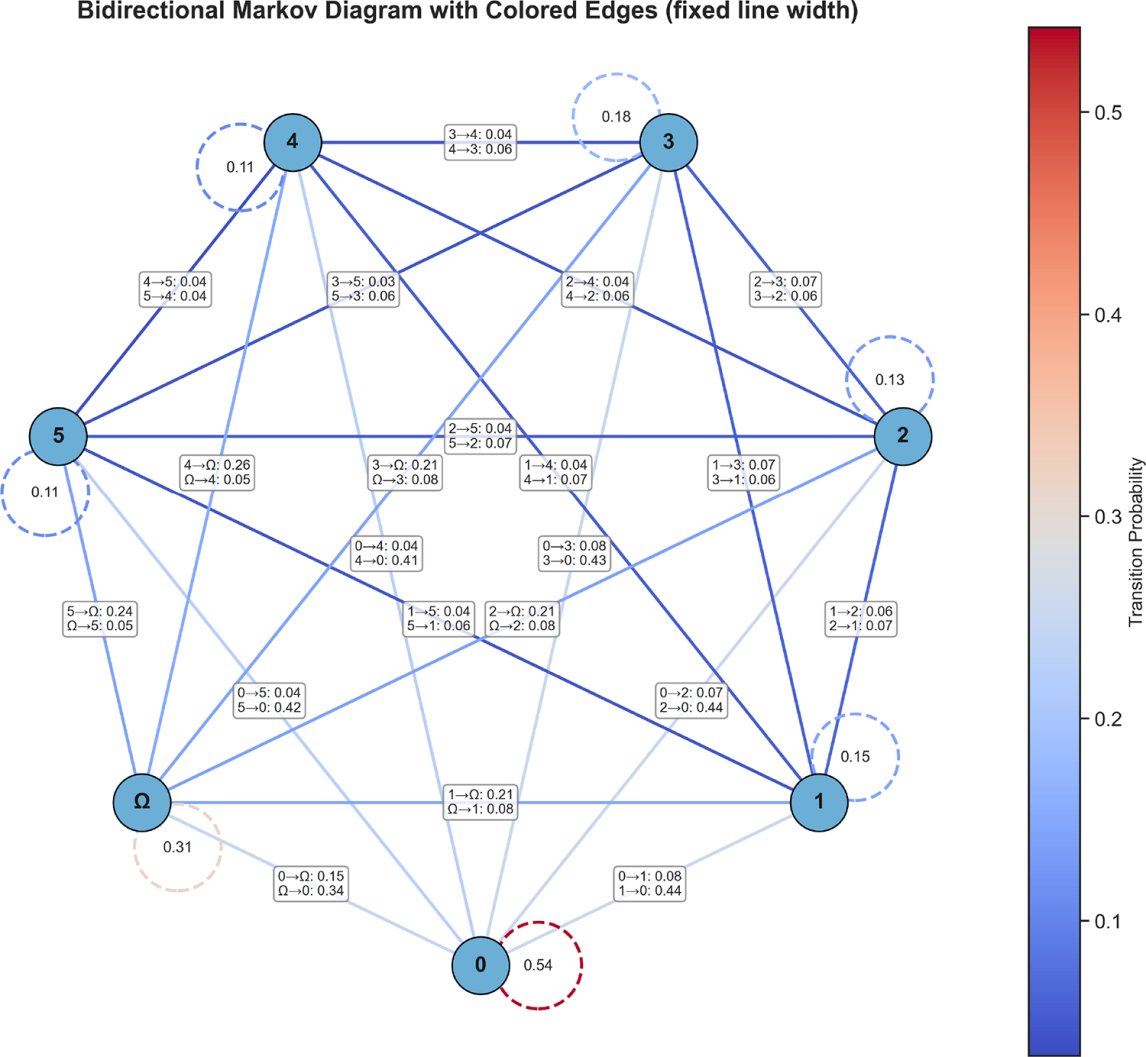
Transition frequency matrix between PCA activation patterns derived from the temporal components. The graph representation illustrates the connectivity between activation patterns and their relative persistence. The baseline pattern and the multicomponent coactivation pattern exhibit the highest persistence, indicating a structured organization of torsional reorganization events along the trajectory.

This behavior suggests a hierarchical organization of torsional reorganizations, in which the system alternates between relatively stable background configurations and transient bursts of coordinated angular activity. The transition structure therefore provides a coarse statistical description of how collective torsional reorganizations propagate along the molecular dynamics trajectory.

To evaluate the robustness of the transition analysis, the transition matrices obtained from the three independent molecular dynamics replicas were compared using three complementary similarity metrics: the Frobenius distance, the Kullback–Leibler (KL) divergence computed across rows, and the Pearson correlation between matrices. The Frobenius norm provides a global measure of the difference between matrices,^17^ the Kullback–Leibler divergence quantifies differences between probability distributions,^18^ and the Pearson correlation coefficient evaluates the linear similarity between the flattened matrices.^19^ The numerical results of these comparisons are summarized in Table 1.

**Table 1:** Pairwise Comparison of Transition Matrices between PCA Activation Patterns Obtained from the Three Independent Molecular Dynamics Replicas^a^

comparison	Frobenius	KL divergence	correlation
R1–R2	0.0278	3.54 × 10^–4^	0.9996
R1–R3	0.0374	8.55 × 10^–4^	0.9992
R2–R3	0.0316	6.72 × 10^–4^	0.9995

a Similarity between matrices is quantified using the Frobenius norm, the row-wise Kullback–Leibler (KL) divergence, and the Pearson correlation coefficient. The very small Frobenius distances and KL divergences, together with correlations above 0.999, indicate that the transition structure of torsional activation patterns is highly reproducible across simulations.

Similarity between matrices is quantified using the Frobenius norm, the row-wise Kullback–Leibler (KL) divergence, and the Pearson correlation coefficient. The very small Frobenius distances and KL divergences, together with correlations above 0.999, indicate that the transition structure of torsional activation patterns is highly reproducible across simulations. The results show extremely high similarity between the transition matrices obtained from independent simulations (Table 1). In particular, the Pearson correlation between matrices exceeds 0.999 in all pairwise comparisons, while the Frobenius distances remain small (≈3 × 10^–2^) and the KL divergences are on the order of 10^–4^.

Figure 9 illustrates the transition matrices obtained from the three replicas. The visual comparison confirms that the dominant transition patterns are preserved across simulations, with consistent high-probability regions and similar overall transition structures.

**Figure 9:**
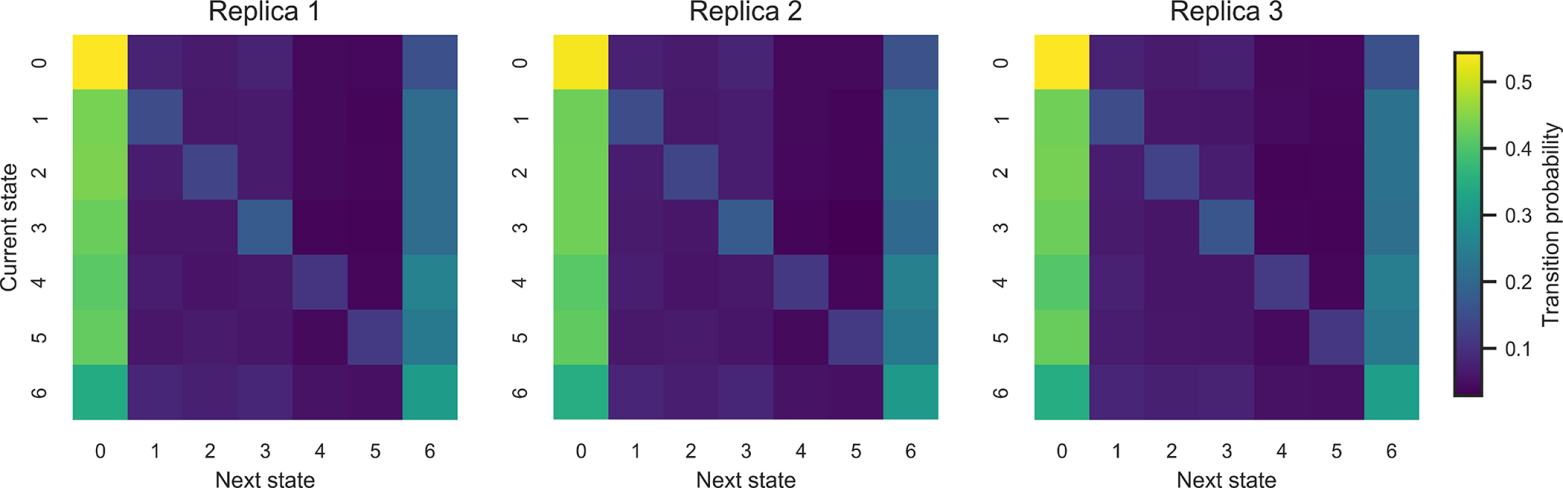
Transition matrices obtained from the three independent molecular dynamics replicas. Each matrix represents the transition frequencies between PCA-mode activation states. The similarity of the matrices across replicas indicates that the statistical organization of the dynamical activity patterns is reproducible.

Taken together, these results demonstrate that the transition structure identified in the PCA activation space is stable across independent simulations. The extremely high correlation between replicas indicates that the dynamical organization of PCA activation patterns is an intrinsic property of the simulated system rather than a sampling artifact. This consistency therefore supports the interpretation that the observed transition structure reflects reproducible features of the peptide’s torsional reorganization dynamics.

We note that this discrete representation of PCA activation patterns does not constitute a conventional kinetic Markov state model (MSM). Traditional MSMs are designed to capture transitions between long-lived metastable conformational states and require validation through implied time scales and Chapman–Kolmogorov tests. In contrast, our aim here is to describe transient bursts of torsional activity—high-frequency reorganization events emphasized by the χ transformation. For this purpose, the reproducibility of the transition patterns across independent replicas (Table 1 and Figure 9) provides the most relevant validation, as it shows that the observed activation dynamics are robust features of the system rather than sampling artifacts.

#### 4.2.4 Results of VAMP

Figure 10 shows the probability distribution of the relative error *e*(*t*) during one-step prediction, evaluated on the validation set for each representation of the dynamics. The distribution associated with the dihedral angles θ exhibits a sharp peak near its mean, indicating that this representation yields, on average, the lowest relative error among all. However, when the PCA-reconstructed trajectories are used as validation data, even lower errors are observed, suggesting that the PCA space captures the most relevant information for structural prediction.

**Figure 10:**
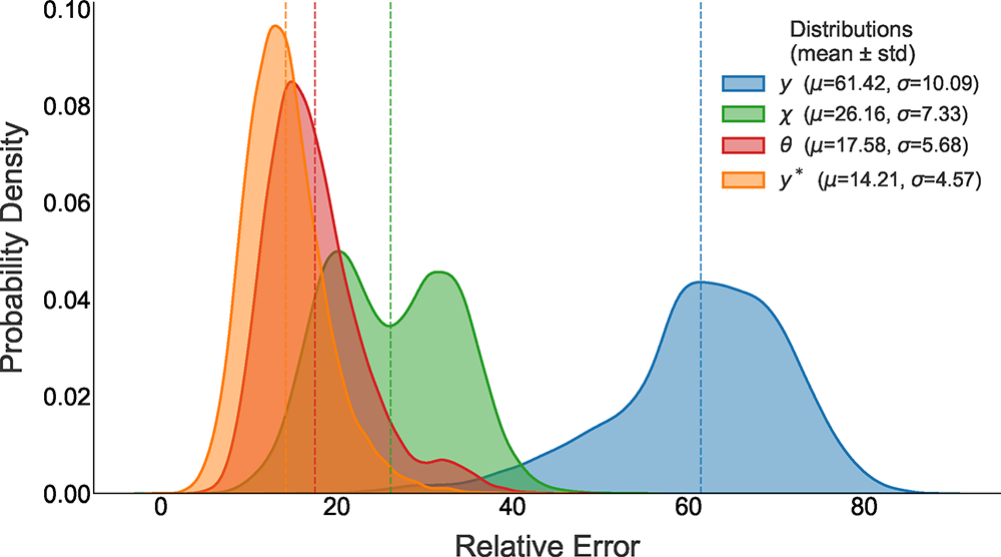
Probability density of the relative prediction error *e*(*t*) for the different dynamical representations of DENV-2: raw dihedral angles (θ), angular displacement (χ), PCA projection (*y*), and trajectories reconstructed from the PCA coordinates (y*). The angular representation (θ) shows the lowest mean prediction error, while the reconstructed PCA trajectories (y*) exhibit an even narrower distribution, indicating that the reduced PCA subspace captures the most relevant dynamical information for short-horizon prediction.

Figure 11 shows the mean prediction error ⟨*e*(τ)⟩ as a function of the lag τ. The lowest average errors correspond to the θ representation, which exhibits a steady increase in error with increasing τ. In contrast, the *y* representation shows an oscillatory pattern in the error, while the χ representation displays a less defined growth, with more irregular variations. These results highlight differences in the predictive stability of each representation as the forecasting horizon increases.

**Figure 11:**
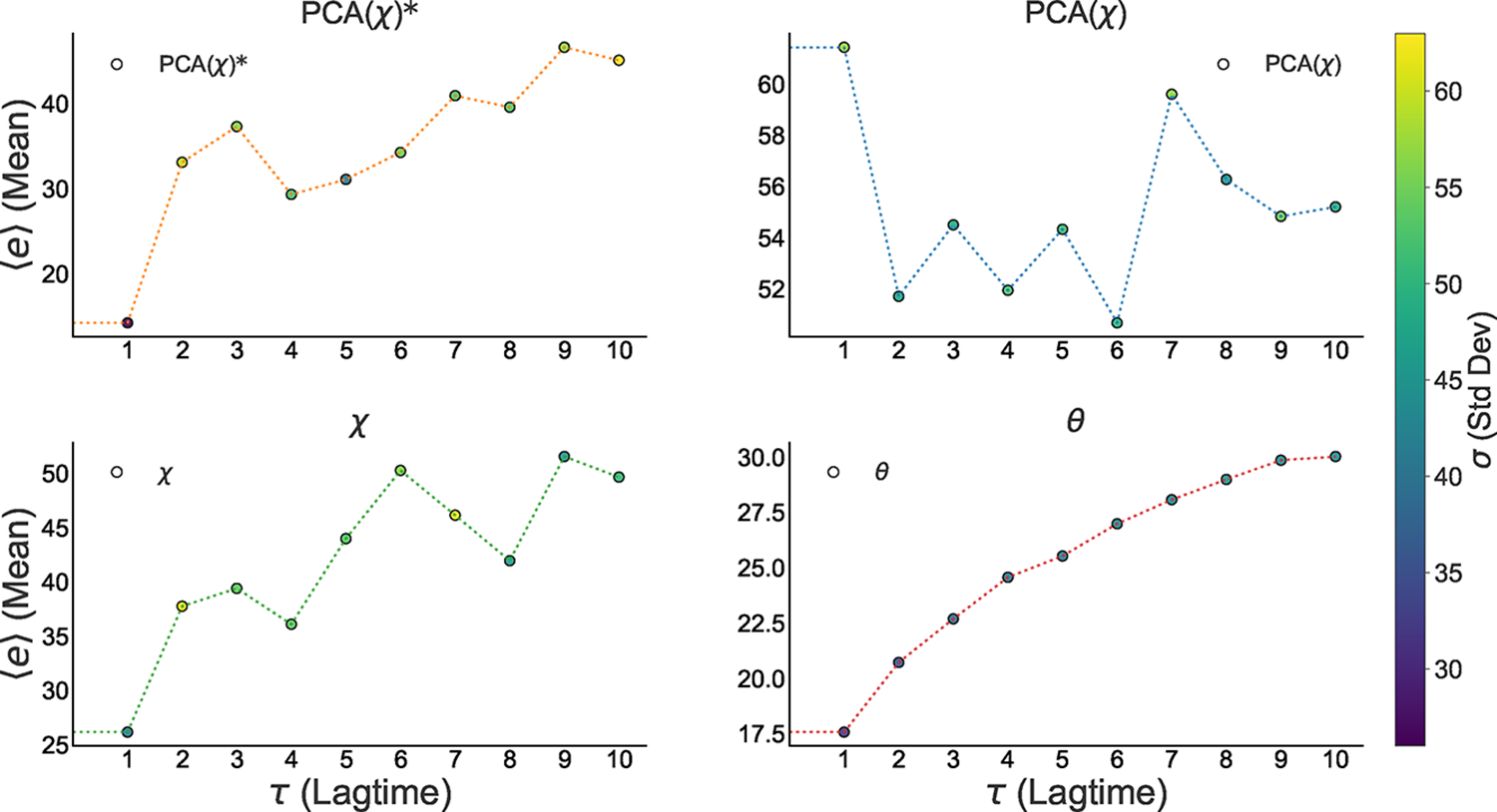
Mean relative error ⟨*e*(τ)⟩ as a function of prediction lag time τ for each representation (*y*, χ, θ). The model based on raw dihedral angles (θ) shows a stable, monotonic increase in error, indicating a predictable decay in accuracy as the time horizon extends. In contrast, the displacement (χ) and PCA (*y*) representations exhibit oscillatory error patterns, suggesting their predictive stability is time scale-dependent and potentially coupled to the specific dynamic modes captured by these coordinates.

Given the relative error defined by the minimum angular metric, it is possible to complement the analysis using a second metric based on atomic coordinates: the RMSD. To this end, the atomic positions must be reconstructed from the predicted dihedral angles. In this way, RMSD serves as a structural metric to quantify the differences between predicted and validation structures (see Figure 12), providing a direct geometric assessment of model performance.

**Figure 12:**
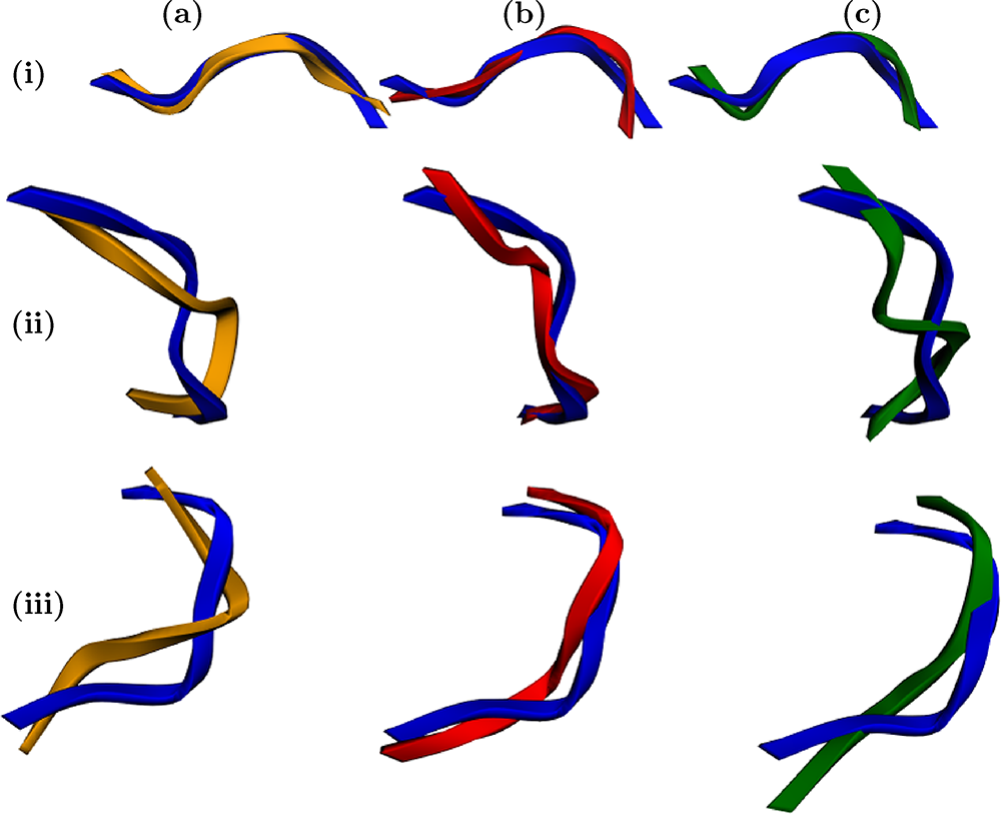
Reconstructed three-dimensional structures of DENV-2. Real conformations are shown in blue, while predictions from the *y*, θ, and χ models are displayed in yellow, red, and green, respectively. The indices (a–c) correspond to the three different predictive functions: *y*, θ, and χ. The indices (i–iii) denote single-step predictions at distinct temporal segments of the validation set: (i) at the beginning, (ii) at the midpoint, and (iii) at the end. Each cell thus represents a specific model–time combination illustrating the structural consistency of the predictions throughout the validation trajectory.

Finally, after reconstructing both the predicted and validation structures, the relative order of the errors remains consistent: the lowest RMSD values correspond to the θ representation, followed by χ, and then *y*. The RMSD values fall within an average range of 1.0–2.14 Å (see Figure 13), indicating acceptable structural fidelity across all cases, with relatively better performance from the raw angular representation.

**Figure 13:**
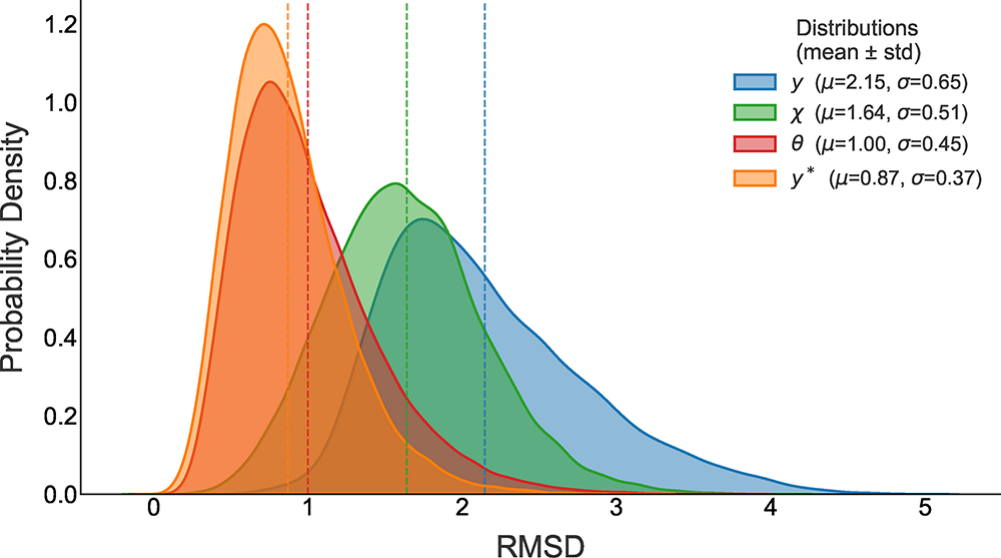
Probability density of the RMSD between predicted and reference DENV-2 structures reconstructed from dihedral (ϕ, ψ, ω) angles for the different dynamical representations: raw dihedral angles (θ), angular displacement (χ), PCA projection (*y*), and trajectories reconstructed from the PCA coordinates (y*). The θ representation yields the lowest RMSD values, while the reconstructed PCA trajectories (y*) exhibit an even narrower distribution, indicating that the reduced PCA subspace captures the most relevant structural information for short-horizon prediction.

### 4.3 Results of the Application of the Method to TRP-CAGE

The TRP-CAGE peptide is used here as a reference ultrafast-folding system to evaluate the predictive capability of the proposed workflow. In this section the system is employed exclusively to test the ability of the methodology to reproduce short-horizon structural evolution using the VAMP predictor, as quantified through angular prediction errors and RMSD reconstruction metrics. The goal is therefore methodological validation of the predictive component of the pipeline rather than a detailed dynamical characterization of the TRP-CAGE conformational landscape.

Due to the higher structural complexity of the TRP-CAGE peptide and the large number of accessible conformational states, the analysis focuses on evaluating the predictive performance of the workflow rather than constructing a detailed transition network. Consequently, the transition matrix analysis used for the DENV-2 system is not included for TRP-CAGE. This choice is consistent with the methodological objective of validating the predictor on a structurally richer reference system.

In addition, the TRP-CAGE trajectory is treated as a benchmark reference data set commonly used in molecular dynamics studies of ultrafast-folding peptides. A standard trajectory is sufficient to evaluate whether the proposed reduced dynamical representation preserves the short-term structural evolution of the system within acceptable prediction-error ranges.

#### 4.3.1 Spacial Components

Figure 14 shows the eigenvalue analysis obtained through PCA applied to the TRP-CAGE system. In Figure 14, it can be observed that the first 20 principal components account for approximately 80% of the total structural variance. This confirms that a large portion of the system’s conformational motion can be effectively described in a low-dimensional reduced subspace. Although no single mode is fully dominant, the collective dynamics can still be characterized by their corresponding explained variance percentages.

**Figure 14:**
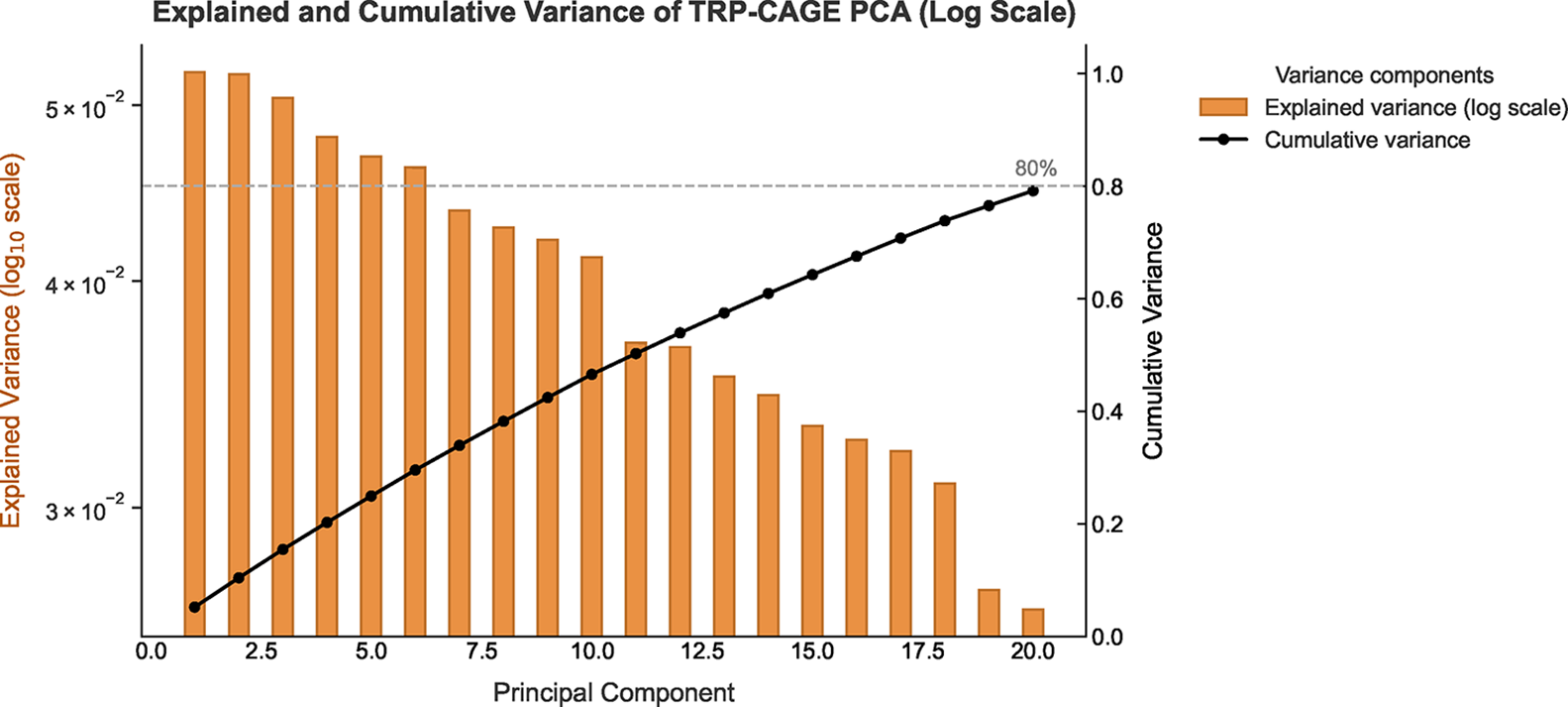
Explained and cumulative variance of the principal components for the TRP-CAGE system. Orange bars represent the individual (log-scaled) variance explained by each component, while the black curve denotes the cumulative variance. The first 20 principal components account for nearly 80% of the total structural variability, illustrating the efficiency of the low-dimensional representation.

Figure 15 shows the distribution of dihedral angle contributions to the first 20 principal components of the TRP-CAGE system. The principal modes are organized according to well-defined structural regions. The first three components, which account for approximately 15% of the total variance, are dominated by collective oscillations centered in the middle region of the peptide (angles 13–20 and 23–28), displaying both coordinated and opposing activation patterns.

**Figure 15:**
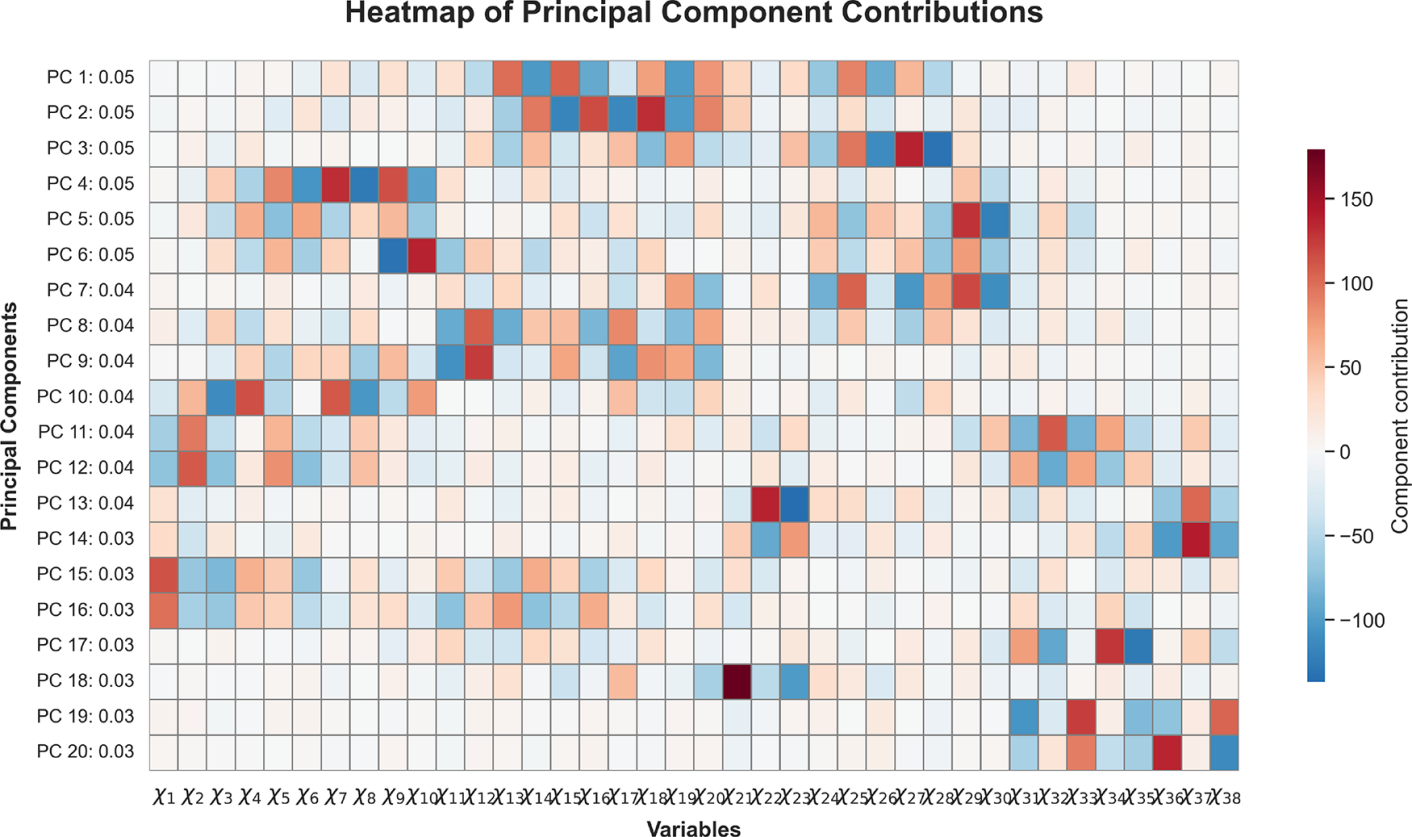
Spatial principal components for the TRP-CAGE peptide. Each mode highlights the dominant dihedral-angle regions contributing to collective motion. The first three components (PC1–PC3) account for approximately 15% of the total variance and are dominated by coordinated oscillations in the central segment (angles 13–20 and 23–28), revealing a highly active oscillatory core. Modes 4–7 capture alternating activations in neighboring torsions (angles 3–11 and 24–30), contributing an additional 19% of the variance, while higher-order modes show more localized activity at the terminal regions (angles 1–6 and 31–38). Overall, the spatial PCA reveals a hierarchical organization of flexibility characterized by a dynamic central core flanked by rigid terminal regions, a hallmark of ultrafast-folding peptides like TRP-CAGE.

Modes 4–7 capture more complex dynamics across angles 3–11 and 24–30, characterized by alternating activations between neighboring angles and low directional synchrony, contributing an additional 19% of the variance. Intermediate modes (e.g., 8 and 9) reflect mixed opposing and aligned movements in the central segment (angles 11–20), while higher-order modes (13–20) show more localized activation at the terminal ends, particularly in angle groups 31–38 and 1–6.

Together, these results reveal a hierarchical organization of angular flexibility in TRP-CAGE: a highly active central oscillatory core flanked by terminal regions with more localized or cooperative motion. This structural architecture aligns with the behavior of ultrafast-folding peptides such as TRP-CAGE, which require a balance between conformational robustness and region-specific flexibility.

#### 4.3.2 Results of VAMP

The predictive experiment on TRP-CAGE therefore serves as an additional validation of the methodology on a structurally more complex peptide. In particular, it allows us to test whether the reduced dynamical representations (θ, χ, and *y*) preserve the short-horizon structural evolution of the system when evaluated through angular metrics and RMSD-based structural reconstruction.

By applying the predictor to the TRP-CAGE peptide, we obtained the distribution of RMSD errors for each system representation (see Figure 16). The errors were comparable across the three representations, ranging from 5.21 to 5.57 Å. In addition, the relative errors remain within a mean interval of 17–34%. As shown in Figure 16, the system representation with the lowest error corresponds to the θ function (red distribution), while the distribution corresponding to the PCA-based predictions with respect to the reconstructed validation data exhibits even lower errors than θ.

**Figure 16:**
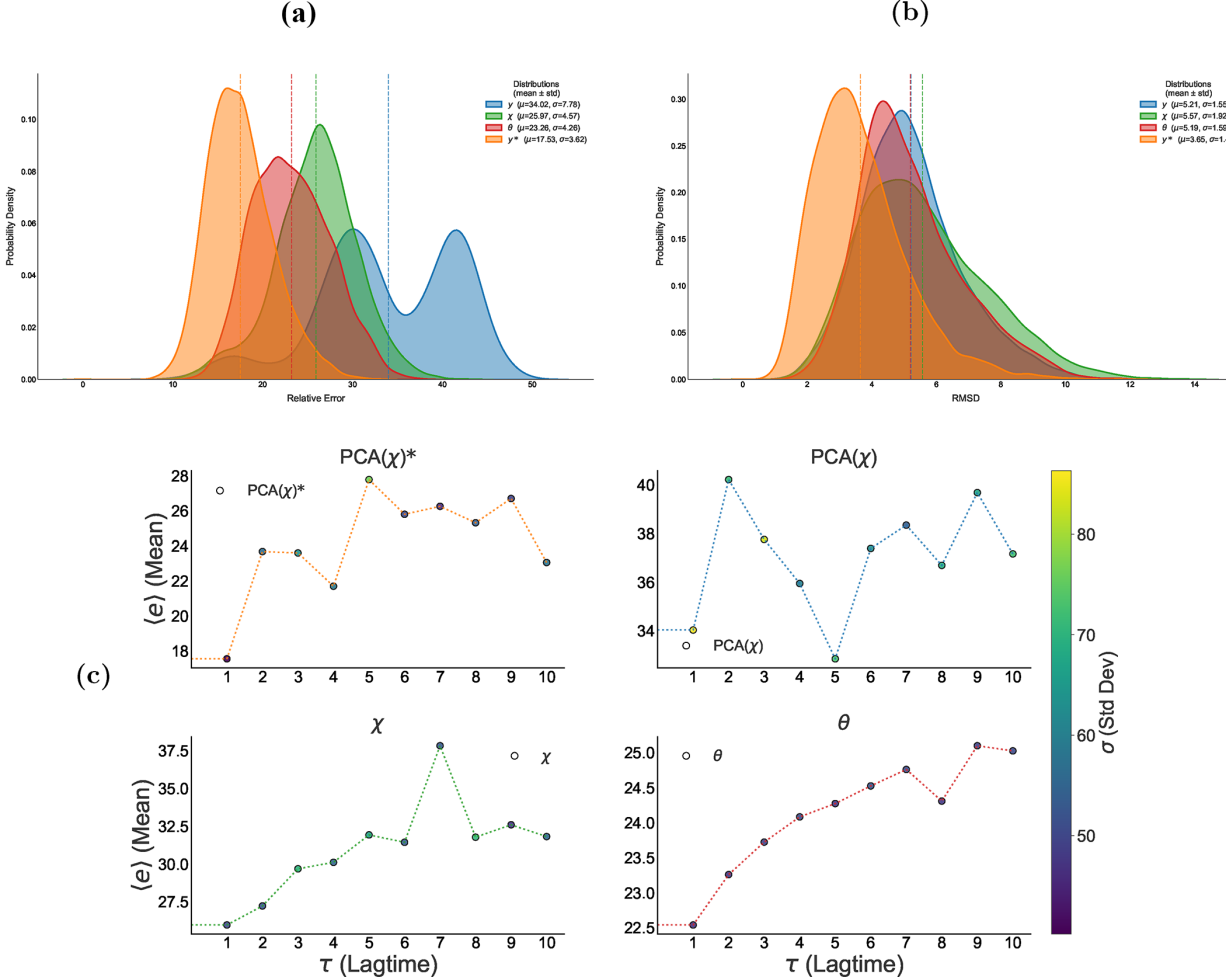
(a) Probability density of the relative prediction error for the different dynamical representations: raw dihedral angles (θ), angular displacement (χ), PCA projection (*y*), and trajectories reconstructed from the PCA coordinates (y*). (b) Probability density of the RMSD between predicted and reference TRP-CAGE structures reconstructed from dihedral (ϕ, ψ, ω) angles. (c) Mean relative prediction error ⟨*e*(τ)⟩ as a function of lag time τ. The angular representation (θ) achieved the lowest average error (RMSD ≈ 5.2 Å, relative error ≈ 23.3*%*) and exhibited a stable increase of ⟨*e*(τ)⟩. In the case of y*, the validation is performed against the angular trajectories reconstructed from the PCA coordinates rather than the original θ trajectory. This comparison isolates the reconstruction error introduced by the PCA dimensionality reduction and allows a direct assessment of the predictive performance in the reduced subspace.

Figure 17 qualitatively illustrates these predictions: the structures generated by the models in PCA space (*y*, yellow), in angular space (θ, red), and in displacement space (χ, green) are compared with the reference (blue). In all cases, the overall topology is preserved, with the θ model showing the highest structural fidelity.

**Figure 17:**
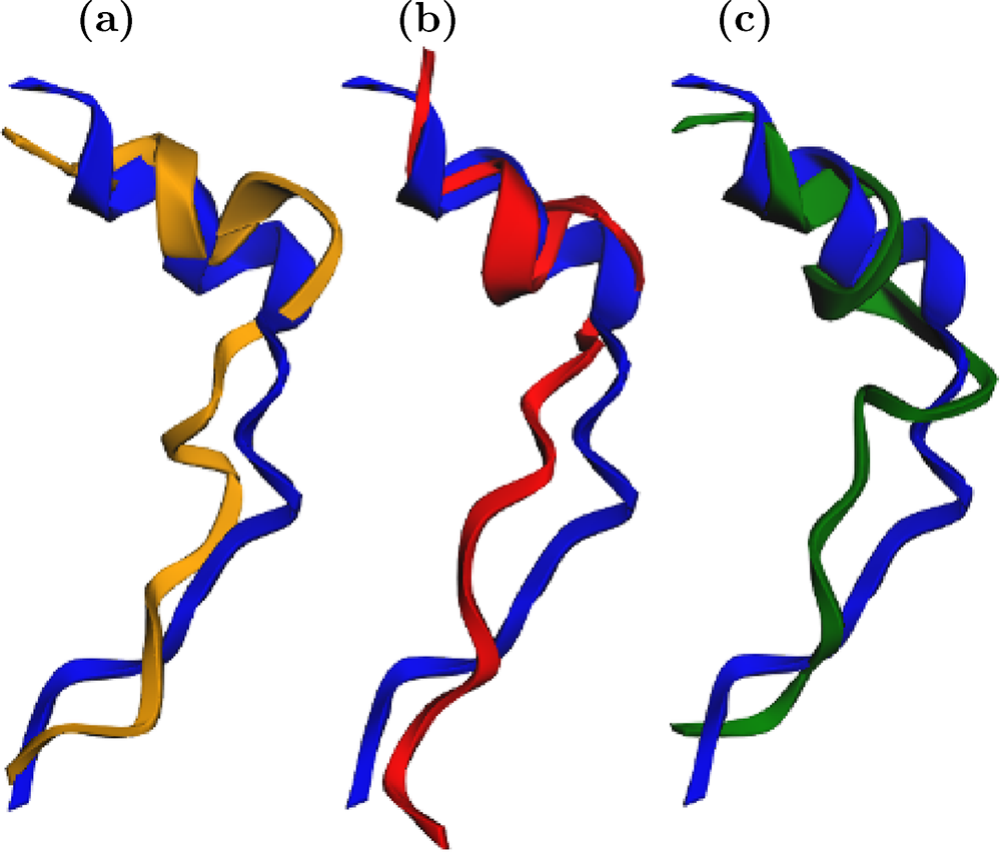
Comparison between predicted and reference TRP-CAGE conformations divided into three panels: (a) prediction in PCA space (*y*, yellow), which captures global collective motions with smooth deformations relative to the reference structure (blue); (b) prediction in angular space (θ, red), which achieves the highest structural fidelity and the lowest RMSD; and (c) prediction in displacement space (χ, green), which preserves torsional continuity and reproduces realistic intermediate conformations. Across all representations, predicted structures remain topologically consistent with the reference, confirming that the Koopman-based VAMP models accurately capture the conformational variability of the ultrafast-folding TRP-CAGE peptide.

## 5 Discussion and Concluding Remarks

Our VAMP-based predictive analysis suggests that the forecasting horizon can be extended while maintaining acceptable structural accuracy. Since each frame corresponds to 2000 integration steps (2000 × 0.002 ps = 4 ps), the lag parameter was increased up to τ = 10, enabling single-step predictions up to 40 ps. This extension allows the model to estimate structural evolution over longer intervals without relying on recursive short-lag predictions, which typically accumulate numerical error.

From a methodological perspective, these results suggest that variational dynamical models based on Koopman operator approximations may offer a useful approach for probing short-horizon predictability in molecular dynamics trajectories.

The spatiotemporal PCA analysis revealed a structured organization of the torsional dynamics of the DENV-2 peptide, where a limited number of principal components capture most of the variance in the angular displacement field. Rather than interpreting these components in biological terms, the analysis highlights how dimensionality reduction can identify coherent patterns of coordinated angular motion along the trajectory. These modes provide a compact description of collective torsional reorganizations and serve as the basis for reduced-order dynamical analysis and prediction.

The predictive reconstruction using VAMP reproduced short-term structural configurations with RMSD values in the range of 1.0–2.1 Å. These results suggest that a reduced dynamical representation of the torsional displacements is sufficient to approximate the short-horizon structural evolution of the system. This predictive capability arises because the combination of angular-displacement observables, PCA dimensionality reduction, and variational dynamical modeling captures the dominant structural fluctuations of the trajectory.

A complementary validation of the predictive component was performed using the TRP-CAGE peptide, a widely used ultrafast-folding benchmark system. In that case, the predictor maintained stable performance with RMSD values around 5.2–5.6 Å and relative angular prediction errors in the range of 17–34*%*, confirming that the reduced dynamical representation preserves short-horizon structural evolution even in a peptide with a more complex conformational landscape.

More broadly, these results illustrate how molecular dynamics trajectories can be analyzed through a combination of geometrically consistent angular representations, dimensionality reduction, and data-driven dynamical modeling. The workflow presented here—angular unwrapping, displacement-based observables χ, spatiotemporal PCA, and VAMP-based prediction—provides a computationally tractable framework for identifying collective torsional reorganizations and assessing short-term predictability in peptide simulations.

The VAMP-2 analysis (Figure 3) shows that the sine–cosine embedding achieves the highest scores, indicating superior preservation of slow dynamical variance. This might suggest that the sine–cosine representation is universally preferable. However, the choice of observable should be guided by the specific dynamical feature of interest. The sine–cosine embedding is indeed well suited for modeling slow conformational kinetics, such as folding transitions between metastable basins. In contrast, the angular displacement representation χ is specifically designed to highlight rapid, transient torsional reorganizations—effectively acting as a discrete angular velocity. Thus, while χ yields lower VAMP scores for slow modes, it is the appropriate choice when the goal is to identify and characterize brief, coordinated torsional events. The two representations are therefore complementary rather than competitive, each serving a distinct analytical purpose.

### 5.1 Limitations and Future Directions

Despite the methodological advantages of the proposed framework, several limitations should be considered. First, the analysis depends on the quality and sampling depth of the molecular dynamics trajectory. Although the 500 ns simulation used here captures the dominant fluctuations of the system, longer simulations or enhanced sampling strategies could reveal additional dynamical regimes not explored in the present data set.

Second, the angular displacement representation χ, while effective for avoiding periodicity artifacts, may introduce distortions when extremely large torsional jumps occur between consecutive frames.^20^ Although such events are rare in the present trajectory, this limitation should be considered when applying the method to systems with highly discontinuous conformational changes.

Third, the predictive component of the framework relies on the assumption that the dominant dynamical information is contained in a reduced subspace obtained from PCA. While this assumption is supported by the variance captured by the leading components in this study, more complex systems may require nonlinear dimensionality reduction techniques or richer feature representations.

Future work could therefore explore: (i) the use of enhanced-sampling trajectories to assess the stability of the identified dynamical modes; (ii) alternative angular representations that preserve geometric continuity while minimizing distortion; and (iii) extensions of the VAMP framework toward multistep prediction schemes capable of maintaining stability over longer forecasting horizons.

### 5.2 Concluding Remarks

In summary, this work presents a computational framework for analyzing torsional dynamics in peptide molecular dynamics simulations through a combination of angular displacement representations, dimensionality reduction, and variational dynamical modeling. The results show that a reduced set of collective modes can capture the dominant fluctuations of the system and provide a suitable basis for short-horizon structural prediction.

Beyond the specific case of the DENV-2 peptide, the methodology illustrates how data-driven analysis can complement traditional molecular dynamics workflows by providing compact representations of high-dimensional trajectories and enabling systematic exploration of dynamical patterns. As such, the proposed approach may serve as a general tool for the statistical characterization and predictive analysis of conformational dynamics in small biomolecular systems.

## Supplementary Material



## Data Availability

For the reproducibility of the Trp-cage dynamics, we provide the exact commands used to download and organize the AMBER tutorial files that served as the initial templates for our protocol setup, which are also available in our GitHub repository. All supplementary data, including simulation files and analysis codes, are available at: https://github.com/Albrizzi/Dengue-antiviral-peptide-DENV-2-structural-analysis-and-prediction-of-molecular-dynamics-simulation.

## Notes

Peptide system prepared in AMBER tleap using the ff19SB protein force field and the OPC water model;^9^ structure (PEP1.pdb) loaded, parametrized, and solvated in an octahedral box of OPC water with a 12 Å buffer; topology and coordinate files generated both in vacuum and in solvent; molecular dynamics simulations carried out in AMBER with sander for minimization and pmemd.cuda for equilibration and production; protocol comprised (i) energy minimization, (ii) two heating stages from 10 to 300 K under restraints, (iii) four equilibration stages at 300 K with progressively reduced restraints, and (iv) production runs in the NPT ensemble at 300 K and 1 atm; and production performed in 10 blocks of 25,000,000 steps each (50 ns per block, 2 fs time step), yielding long trajectories in NetCDF format for subsequent analysis (PDF)
