# Supplementary material for: Analyzing Peptide Torsional Dynamics: An Angular-Displacement PCA Pipeline for Short-Horizon Prediction from Molecular Dynamics
Source: J Phys Chem A. 2026 Apr 22;130(18):3665–79. doi: 10.1021/acs.jpca.5c07760 (PMC13158914; doi:10.1021/acs.jpca.5c07760)
Supplement: Supplementary file 1 [file jp5c07760_si_001.pdf]

Analyzing Peptide Torsional Dynamics: An Angular-Displacement PCA Pipeline for Short-Horizon Prediction from Molecular Dynamics

Luis Albrizzi†, Gabriel Gayoso†, José Colbes†, Santiago Di Lella *◇, Christian E. Schaerer††, and Amaury C. Alvarez‡⋆

†Polytechnic School, National University of Asuncion, Campus de la UNA, Villa Universitaria, San Lorenzo 111421, Central, Paraguay. P.O.Box: 2111 SL.
◇Instituto de Química Biológica - Ciencias Exactas y Naturales (IQUIBICEN) Conicet - Facultad de Ciencias Exactas y Naturales, Universidad de Buenos Aires, Ciudad Universitaria, Pab. II, C1428EHA Ciudad de Buenos Aires, Argentina
⋆Institute of Computing, Federal University of Rio de Janeiro, Av. Athos da Silveira Ramos, 274, Rio de Janeiro, 21941-590, Rio de Janeiro, Brazil.

*Email: amaury@ic.ufrj.br

Supporting Information

S1.1 Modular projection and lifting (unwrapping)

Let $\{\theta_t\}_{t=0}^{T} \subset \mathbb{T}^n \cong [-\pi,\pi)^n$ be a sequence of angular vectors (e.g., dihedral angles) observed along a trajectory. Because each coordinate is periodic modulo 2π, direct differences $\theta_t - \theta_{t-1}$ may contain artificial jumps at the $\pm\pi$ boundary. To remove these discontinuities, we define a modular projection

$$M(\alpha) \;=\; (\alpha+\pi) \bmod 2\pi - \pi \quad \in [-\pi,\pi), \tag{1}$$

and extend it componentwise to vectors: $M(x) = (M(x^{(1)}),\ldots,M(x^{(n)}))$.

Using M, we construct the lifted (unwrapped) trajectory $\gamma_t \in \mathbb{R}^n$ componentwise as

$$\gamma_t^{(j)} = \begin{cases} \theta_0^{(j)}, & t=0, \\ \gamma_{t-1}^{(j)} + M\Big(\theta_t^{(j)} - \theta_{t-1}^{(j)}\Big), & t \ge 1, \end{cases} \qquad j = 1,\ldots,n. \tag{2}$$

This lifting preserves the angular information while producing a continuous representative in the universal covering space $\mathbb{R}^n$.

S1.2 Reversibility of the lifting

Proposition S1 (Reversibility). For all $t = 0,\ldots,T$, the lifting satisfies

$$M(\gamma_t) = \theta_t. \tag{3}$$

*Email: santiagodilella@gmail.com
†Email: fernandoalbrizzi89@fpuna.edu.py, gayosogabs00@fpuna.edu.py, {jcolbes, cschaer}@pol.una.py
‡Corresponding Author Email: Email: amaury@ic.ufrj.br

Proof. We prove the statement by induction on t.

Base case ($t = 0$). By definition, $\gamma_0 = \theta_0$ and since $\theta_0 \in [-\pi, \pi)^n$,

$$M(\gamma_0) = M(\theta_0) = \theta_0.$$

Inductive step. Assume $M(\gamma_t) = \theta_t$ for some $t \geq 0$. By the definition of M, the equality $M(\gamma_t) = \theta_t$ implies that γ_t and θ_t are equivalent modulo 2π componentwise. Therefore, there exists an integer vector $k \in \mathbb{Z}^n$ such that

$$\gamma_t = \theta_t + 2\pi k.$$

Using the lifting recurrence,

$$\gamma_{t+1} = \gamma_t + M(\theta_{t+1} - \theta_t) = \theta_t + 2\pi k + M(\theta_{t+1} - \theta_t).$$

Now note that $M(\theta_{t+1} - \theta_t)$ is exactly the representative of the increment $(\theta_{t+1} - \theta_t)$ in $[-\pi, \pi)^n$, so there exists an integer vector $k' \in \mathbb{Z}^n$ such that

$$M(\theta_{t+1} - \theta_t) = (\theta_{t+1} - \theta_t) - 2\pi k'.$$

Substituting,

$$\gamma_{t+1} = \theta_t + 2\pi k + (\theta_{t+1} - \theta_t) - 2\pi k' = \theta_{t+1} + 2\pi(k - k').$$

Hence γ_{t+1} and θ_{t+1} are equivalent modulo 2π, and applying M yields

$$M(\gamma_{t+1}) = \theta_{t+1}.$$

This completes the induction. □

S1.3 Definition of the angular displacement χ

Once γ_t is defined, the angular displacement is

$$\chi_t := \gamma_t - \gamma_{t-1}, \qquad t \geq 1. \tag{4}$$

Using the definition of γ_t, this can be written equivalently as

$$\chi_t = M(\theta_t - \theta_{t-1}), \tag{5}$$

so χ_t corresponds to the *minimal (geodesic) increment* between consecutive angular configurations, free of wrapping artifacts.

S1.4 Geometric meaning

The original variables θ_t evolve on the torus $\mathbb{T}^n$. The lifting γ_t is a trajectory in the universal cover $\mathbb{R}^n$ such that projecting back with M recovers the original angles. The displacement χ_t therefore lives naturally in a linear space and can be interpreted as an element of the tangent representation associated with the lifted trajectory.

S1.5 Physical Interpretation of the Angular Displacement

The variable $\chi(t)$ can be interpreted as a **discrete angular velocity**:

$$\chi(t) \approx \Delta t\, \dot{\theta}(t), \tag{6}$$

which is valid when Δt is small. The implications are:

- **Small values** of $|\chi_i(t)|$ indicate **local structural persistence**.
- **Large values** indicate a **rapid reorganization** of that angle (a change "event").
- Simultaneous activations across multiple angles indicate **coordinated collective reorganization**.

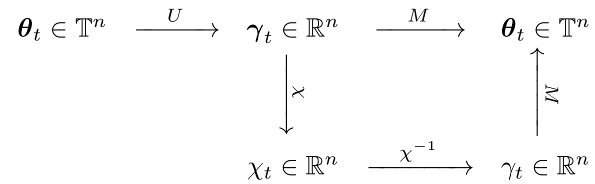

Figure 1: Complete diagram of the transformation pipeline including prediction pathways. The diagram shows the three alternative routes for VAMP-based prediction: K_θ acting directly on θ_t, K_χ acting on χ_t, and K_y acting on y_t. Predicted quantities (hat) are transformed back to angular space via the appropriate inverse mappings (χ^{-1}, M, V^T). This figure extends the basic pipeline of Figure S1.

S2. Spatiotemporal PCA representation

To characterize collective torsional reorganizations, we analyze the angular-displacement observables $\chi(t) \in \mathbb{R}^n$ using principal component analysis (PCA). Let $X \in \mathbb{R}^{T\times n}$ denote the data matrix obtained by stacking the observations $\chi(t)$ along time. After centering the data by column, the matrix can be written as

$$H = X - \mathbf{1}\mu^T,$$

where μ is the vector of column means.

The spatiotemporal decomposition is obtained through the singular value decomposition (SVD):

$$H = U\Sigma V^T,$$

where $V \in \mathbb{R}^{n\times r}$ contains the spatial principal components, $U \in \mathbb{R}^{T\times r}$ contains the corresponding temporal modes, and $\Sigma = \mathrm{diag}(\sigma_1, \dots, \sigma_r)$ are the singular values.

This decomposition provides an exact separation between spatial and temporal structures:

$$H = \sum_{k=1}^{r} \sigma_k \, u_k v_k^T,$$

where v_k represents a spatial pattern of coordinated torsional reorganization and u_k describes its temporal activation along the trajectory.

The original data can therefore be reconstructed as

$$X = \mathbf{1}\mu^T + \sum_{k=1}^{r} \sigma_k \, u_k v_k^T.$$

In practice, only the first K modes are retained,

$$X \approx \mathbf{1}\mu^T + \sum_{k=1}^{K} \sigma_k \, u_k v_k^T,$$

which provides the optimal rank-K approximation in the Frobenius norm (Eckart–Young–Mirsky theorem). The retained spatial modes identify the dominant patterns of collective torsional reorganization, while the temporal coefficients quantify when and how strongly these patterns are activated along the molecular dynamics trajectory.

S3. Reduced dynamics in PCA space (Propositions 2 and 3)

Proposition 2 (EDMD-based reduction). Let $\chi_t \in \mathbb{R}^n$ be the observable and $V \in \mathbb{R}^{n\times r}$ the matrix of orthonormal PCA directions. If a linear operator $K^* \in \mathbb{R}^{n\times n}$ satisfies $\dot{\chi}_t \approx \chi_t K^*$ (in continuous time) or $\chi_{t+1} \approx \chi_t K^*$ (discrete), then the projected variable $y_t = \chi_t V$ evolves approximately as $y_{t+1} = y_t M^*$ with $M^* = V^\mathsf{T} K^* V$. The proof follows directly from multiplying the evolution equation by V and using $\chi_t = y_t V^\mathsf{T}$.

Proposition 3 (VAMP-based reduction). If the Koopman operator K satisfies $\mathbb{E}[\chi_{t+1}] = \mathbb{E}[\chi_t]K$, then $\mathbb{E}[y_{t+1}] = \mathbb{E}[y_t]K_y$ with $K_y = V^{\mathsf{T}}KV$. The proof is analogous, taking expectations and using the same projection.

These relations show that any linear model in the original observable space induces a consistent linear model in the PCA subspace.

S4. Additional details on state discretization and threshold selection

The threshold ϵ for each temporal component y_k is chosen as the $(100 - \lambda_k)$-th percentile of $|y_k|$, where λ_k is the percentage of variance explained by that component. This ensures that the most energetic fluctuations are retained while noise is suppressed. For DENV-2, we used $\lambda_k = 80\%$ cumulative variance, leading to thresholds around 1.5–2.0 (arbitrary units). The stability of the resulting Markov model with respect to ϵ was verified; results reported in the main text correspond to the median ϵ over all components.

S5. Detailed transformation diagrams

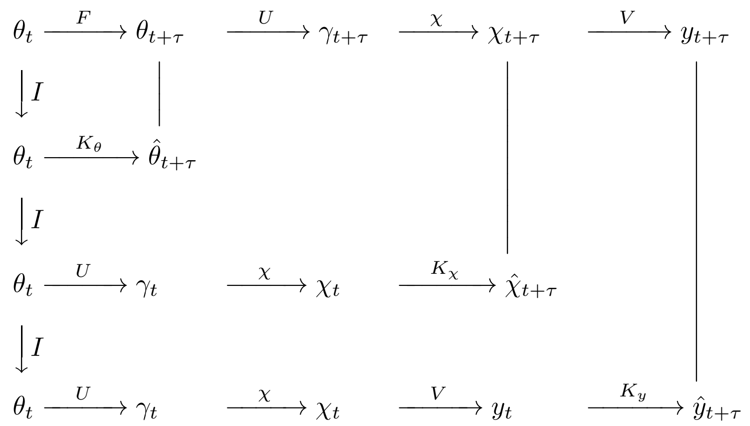

Figure 2: Schematic of the transformation pipeline for dihedral angles. The original wrapped angles θ_t are unwrapped (U) to continuous coordinates γ_t, from which the displacement χ_t is computed. The inverse mapping χ^{-1} reconstructs γ_t, and the modular projection M returns to θ_t. This reversible pipeline enables linear analysis on χ_t while preserving the ability to recover the original angles.

S6. Formal Derivation in the Fourier Domain (High-Pass Behavior)

Let $\gamma[n]$ denote the discrete sequence (with $t = n\Delta t$) and $\Gamma(\omega)$ its Fourier transform:

$$\Gamma(\omega) = \sum_{n=-\infty}^{\infty} \gamma[n]\, e^{-i\omega n \Delta t}. \tag{7}$$

The discrete difference $\chi[n] = \gamma[n] - \gamma[n-1]$ has Fourier transform:

$$X(\omega) = \left(1 - e^{-i\omega\Delta t}\right)\Gamma(\omega), \tag{8}$$

and

$$|1 - e^{-i\omega\Delta t}|^2 = 4\sin^2\left(\frac{\omega\Delta t}{2}\right). \tag{9}$$

As $\omega \to 0$ (slow components), the multiplicative factor tends to zero, suppressing low-frequency contributions. For higher frequencies, the factor increases in magnitude. Therefore, $\chi(t)$ behaves as a **high-pass filter**, emphasizing rapid or transient reorganizations while attenuating slow drift.

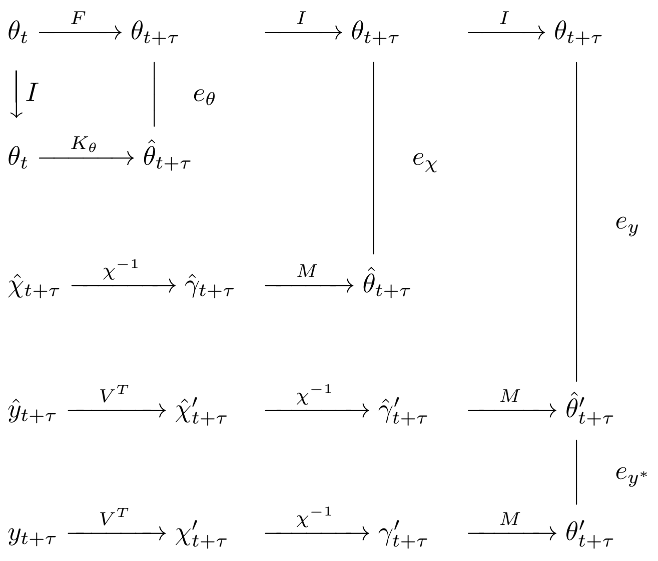

Figure 3: Flow diagram illustrating the computation of angular-space error vectors (e_θ, e_χ, e_y, e_{y^*}). For each representation, the VAMP prediction is reconstructed to angular coordinates and compared with the true angles using the angular metric defined in the main text. This ensures that predictions from different representations are evaluated on a common basis.

This property explains why χ is not designed to maximize slow modes, but rather to capture reorganization events.
